# Molecular and cellular dynamics of early embryonic cell divisions in *Volvox carteri*

**DOI:** 10.1093/plcell/koac004

**Published:** 2022-01-09

**Authors:** Eva Laura von der Heyde, Armin Hallmann

**Affiliations:** Department of Cellular and Developmental Biology of Plants, University of Bielefeld, Universitätsstr. 25, 33615 Bielefeld, Germany

## Abstract

Cell division is fundamental to all organisms and the green alga used here exhibits both key animal and plant functions. Specifically, we analyzed the molecular and cellular dynamics of early embryonic divisions of the multicellular green alga *Volvox carteri* (Chlamydomonadales). Relevant proteins related to mitosis and cytokinesis were identified in silico, the corresponding genes were cloned, fused to *yfp*, and stably expressed in *Volvox*, and the tagged proteins were studied by live-cell imaging. We reveal rearrangements of the microtubule cytoskeleton during centrosome separation, spindle formation, establishment of the phycoplast, and generation of previously unknown structures. The centrosomes participate in initiation of spindle formation and determination of spindle orientation. Although the nuclear envelope does not break down during early mitosis, intermixing of cytoplasm and nucleoplasm results in loss of nuclear identity. Finally, we present a model for mitosis in *Volvox*. Our study reveals enormous dynamics, clarifies spatio-temporal relationships of subcellular structures, and provides insight into the evolution of cell division.

## Teaser

Analysis of cell divisions of the microalga *Volvox* reveals enormous dynamics of cytoskeletal and membranous structures with coordination of intranuclear spindle formation by cytosolic centrosomes.

IN A NUTSHELL **Background:** Mitosis, a type of cell division, is fundamental to all eukaryotic life and must be carried out very accurately. Even though the process of mitosis itself is highly conserved among eukaryotes, there are significant differences between animals, fungi, plants, and algae. From an evolutionary point of view, the green alga *Volvox carteri* used here possesses both key animal and plant functions and it exhibits important features of the last common eukaryotic ancestor that have been lost in other lineages. Prior to our work, a comprehensive in vivo analysis of the entire process of cell division in green algae was lacking.

**Question:** How exactly does cell division work in green algae? How do the cytosolic centrosomes deal with the persistent nuclear envelope in this process? What is the relationship between different microtubular structures?

**Findings:** Our study reveals enormous dynamics during mitosis, clarifies spatio-temporal relationships of subcellular structures, and provides insights into evolution of cell division. Although the nuclear envelope does not break down during early mitosis of *Volvox*, it becomes permeable and the nucleus temporarily loses its identity. Two microtubule-organizing centers, the centrosomes, located immediately outside the nuclear envelope participate in initiation of the mitotic spindle formation inside the nuclear envelope. This process also defines the orientation of the mitotic spindle. In cytokinesis, an algae-specific microtubule structure, the phycoplast, replaces the spindle. The microtubules of the phycoplast may play a direct role in promoting the cell membrane invagination of the cleavage furrow.

**Next steps:** How are the massive rearrangements of subcellular structures regulated? What happens at the nuclear pores when the nuclear envelope becomes permeable at the onset of mitosis? What determines in later embryogenesis which cells then divide asymmetrically rather than symmetrically?

## Introduction

Cell division is one of the most fundamental processes of life. Even though the mitotic process itself is highly conserved among eukaryotes, there are significant differences in this process between animals, fungi, plants, and algae (Drechsler and McAinsh, 2012). Such differences exist, for example, in the structure and organization of the cytoskeleton, which is substantially involved in the process (Joukov and De Nicolo, 2019). Animal cells mostly use centrosomes to orchestrate microtubular structures and a contractile actin/myosin ring to form the cleavage furrow (Cheffings et al., 2016; Petry, 2016). In contrast, vascular plants, which do not have centrosomes, form plant-specific microtubular structures such as the preprophase band and the phragmoplast, which serve to define the division plane and to guide cytokinesis, respectively (Buschmann and Zachgo, 2016). There are also major differences between eukaryotes in terms of how to handle the nuclear envelope during cell division. While there is usually a temporary breakdown of the nuclear envelope in animal and vascular plant mitosis, called “open mitosis,” most fungi perform “closed mitosis” in which the nuclear–cytoplasmic compartment barrier remains largely intact throughout the division process. The microtubule cytoskeleton of many fungi is organized by spindle pole bodies, which are embedded in the nuclear envelope and nucleate cytoplasmic microtubules as well as spindle microtubules (Winey and O’Toole, 2001). However, there are also examples of non-centrosomal microtubule-organizing centers (MTOCs) in fungi (Hagan, 1998; Zhang et al., 2017).

**Figure 1:**
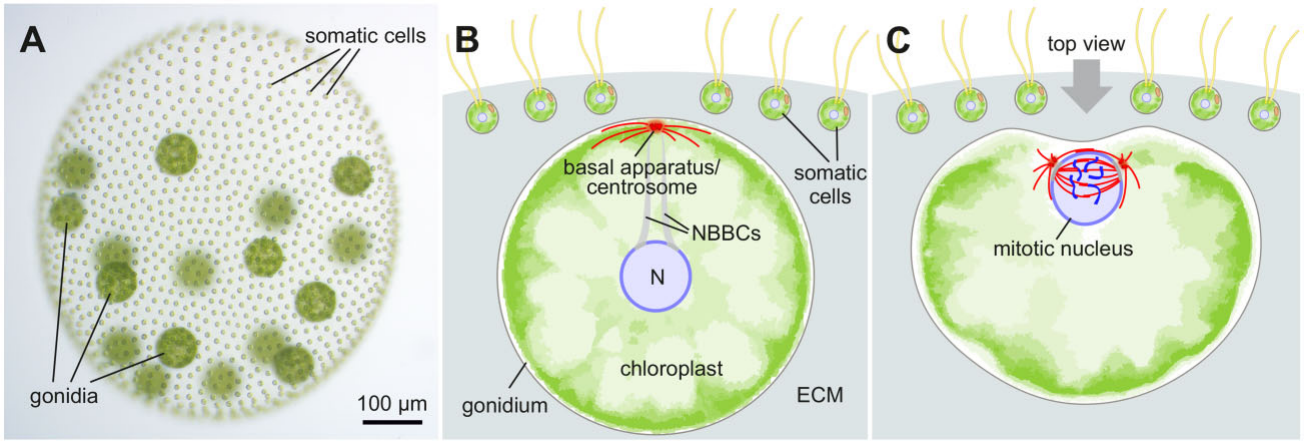
Phenotype and schematic cross-section of *V. carteri*. A, Wild-type phenotype of an asexual, female *V. carteri* spheroid containing approximately 2000 small, terminally differentiated, biflagellate somatic cells at the surface and approximately 16 large reproductive cells just below the somatic cell layer. The gonidia are at the stage immediately before the beginning of the first embryonic cell division. A transparent, glycoprotein-rich ECM holds the cells in place and constitutes up to 99% of the volume of the spheroid. B and C, Schematic cross section of a part of the *V. carteri* spheroid illustrating the arrangement of the cells and relevant subcellular structures. A gonidium is depicted at two mitotic stages in side view. The gonidial nucleus is located in the center of the cell during interphase, but comes close to the cell membrane during preprophase. This provides an unobstructed view from the outside of the spheroid onto the dividing nucleus. B, Gonidium during interphase. N, nucleus. C, Gonidium during metaphase of the first embryonic division. The nucleus comes close to the cell membrane during preprophase. Gray arrow indicates the most suitable viewing direction (top view) for the observation of mitosis.

In the large group of algae (including green, red and brown algae, diatoms, and dinoflagellates), the diversity and frequency of occurrence of different forms of mitosis can currently only be described incompletely, since mitosis has only been studied in relatively few species (Pickett-Heaps, 1976; Buschmann and Zachgo, 2016). However, it is already clear that there are not only open and closed types of mitosis in this group, but also various intermediate forms as seen in many other eukaryotes (Makarova and Oliferenko, 2016). Of particular interest is the detailed study of mitosis of green algae, as ancestral green algae represent the starting point for the evolutionary transition to the earliest land plants (Graham, 1996). Most of the information on mitosis of green algae (Chloroplastida, Chlorophyta) comes from the unicellular, biflagellate microalga *Chlamydomonas reinhardtii* (Marshall and Rosenbaum, 2000; Harris, 2001; Harris et al., 2009), a member of the volvocine algae within the Chlamydomonadales. In this study, we focus on mitosis of the multicellular volvocine alga, *V.* *carteri* (Figure 1A), a quite close relative of *C. reinhardtii*. An argument for explicitly using the green algae *Chlamydomonas* or *Volvox* in the study of mitosis is that these algae have important features of the last eukaryotic common ancestor (LECA) that have been lost in other lineages (Cross and Umen, 2015). Interestingly, volvocine algae show common characteristics with both vascular plants and animals (Merchant et al., 2007).

In contrast to *C. reinhardtii*, *V. carteri* shows a germ-soma differentiation with two distinct cell types: in its asexual phase, it possesses approximately 2000 small, biflagellate, terminally differentiated somatic cells, which form a monolayer at the surface of a sphere, and approximately 16 large, flagella-less reproductive cells (gonidia), which lie just below the sheet of somatic cells (Figure 1B). All of the cells are regularly positioned within a transparent glycoprotein-rich extracellular matrix (ECM) that occupies up to 99% of the spheroid volume (Figure 1A). The large size of the gonidia is due to the fact that in *V. carteri* and most other volvocine algae cell divisions occur by palintomy (rapid multiple fission) (Coleman, 1979; Sleigh, 1989; Desnitski, 1995; Herron et al., 2010), that is the gonidia first grow strongly and then undergo a rapid sequence of repeated divisions without intervening growth. In this process, the first five cell divisions are symmetrical and an embryo with 32 cells of the same size is formed (Kirk, 1998; Kirk and Kirk, 2004; Hallmann, 2011). Then 16 cells in one hemisphere divide asymmetrically to produce one large gonidial cell initial and one small somatic cell initial each. The gonidial initials divide asymmetrically two more times and produce additional somatic initials at each division. The gonidial initials then temporarily stop any cleavage activity, while the somatic initials divide symmetrically about three more times. The embryo then contains all cells that will be present in an adult of the next generation but the orientation of the cells is not yet correct, that is the cells have their ﬂagellar ends pointing toward the interior, rather than toward the exterior where they will be needed to function in locomotion. To achieve the correct adult configuration, the embryo turns itself right-side out in a gastrulation-like morphogenetic process called (embryonic) inversion (Kirk, 1998; Hallmann, 2006b). The resulting juveniles expand through the deposition of ECM and then hatch from their mother spheroid.

Previous research in *Volvox* mitosis is limited to a few, older studies using fixed cells and only a few time points (Deason and Darden, 1971; Birchem and Kochert, 1979; Hoops, 1984; Kirk et al., 1991). In *V. carteri* f. *weismannia*, the structure of the basal body apparatus of somatic cells is similar to that in *Chlamydomonas* (Hoops, 1984). In *V. aureus* sperm precursor cells, microtubules extend between the pairs of basal bodies as they migrate apart during mitosis (Deason and Darden, 1971). In sperm precursor cells of *V. carteri* f. *weismannia*, microtubules radiate from an area of granular material near the basal bodies (Birchem and Kochert, 1979). Furthermore, the nucleus in mitosis changes its position and an algae-specific microtubule structure, the phycoplast, forms in cytokinesis (Birchem and Kochert, 1979). In *V. carteri* f. *nagariensis*, there were indications that basal body separation precedes spindle formation and that the nuclear envelope persists throughout mitosis (Kirk et al., 1991). It should also be noted that the fixed cells used in previous work precluded further tracking of a particular cell and its division products. Overall, comprehensive in vivo analyses of not only the microtubule cytoskeleton but the entire process of mitosis in *Volvox* have been lacking so far. Instead, multicellular volvocine algae have been used primarily to study embryogenesis, cellular differentiation, morphogenesis, and ECM biogenesis as well as the evolution of these developmental processes and multicellularity in general (Kirk, 1998; Hallmann, 2003; Kirk, 2005; Hallmann, 2006a, 2011; Herron, 2016).

Much more is known of the mitosis in the green microalga *Chlamydomonas*, the unicellular relative of *Volvox*. In *Chlamydomonas*, each cell contains a basal apparatus/centrosome, which is very similar to the corresponding metazoan organelle (Marshall and Rosenbaum, 2000; Pazour and Witman, 2009) and accordingly serves as the main MTOC of the cell (Doonan and Grief, 1987). The basal apparatus of *C. reinhardtii* includes a pair of basal bodies, which nucleate the flagella (Dutcher and O’Toole, 2016; Wingfield and Lechtreck, 2018). Each mature basal body/centriole of *Chlamydomonas* is associated with a pair of microtubular rootlets, which are involved in positioning of intracellular structures, determination of the division plane, and centrosome separation during mitosis (Holmes and Dutcher, 1989; Ehler and Dutcher, 1998). Replication and elongation of the basal bodies/centrioles have been extensively studied in *C. reinhardtii* (Gould, 1975; Gaffal, 1988; O’Toole and Dutcher, 2014), whereas less is known about the timing and mechanism of centrosome separation, which duplicates the cell’s central MTOC early in mitosis. This movement is guided by cytoskeletal structures (Holmes and Dutcher, 1989) and positions the duplicated centrosomes of *Chlamydomonas* on opposite sides of the nucleus near where the spindle poles will be located (Johnson and Porter, 1968; Coss, 1974). Similar to the nuclear envelope of fungi, the nuclear envelope of the green alga *Chlamydomonas* does not break down before spindle formation. Centrosomes of *C. reinhardtii* are therefore thought to induce and coordinate spindle formation through polar fenestrae in the nuclear envelope (Johnson and Porter, 1968; Coss, 1974; Birchem and Kochert, 1979; Doonan and Grief, 1987; O’Toole and Dutcher, 2014). Not before the end of anaphase, the nuclear envelope of *C. reinhardtii* breaks down and reforms around the chromatin of the emerging daughter cells by utilization of the previous envelope components (Johnson and Porter, 1968). In the course of cytokinesis of *Chlamydomonas*, a microtubule array forms at the division plane, the phycoplast, which is distinctly different from the phragmoplast of vascular plants (Marshall, 2009). It has been postulated that the phycoplast ensures that the plane of cell division passes exactly between the two daughter nuclei and that the microtubules of the phycoplast may play a direct role in promoting furrow ingression in *C. reinhardtii* (Ehler and Dutcher, 1998; O’Toole and Dutcher, 2014). Later, the involvement of actin and myosin-based structures in the formation of the cleavage furrow was also investigated in *Chlamydomonas*, but could then be excluded experimentally (Onishi et al., 2020). Therefore, exploring the role of microtubules in furrow formation was set as a major goal of future studies (Onishi et al., 2020). However, visualization of microtubules by time-lapse imaging using a fluorescently tagged tubulin failed in *C. reinhardtii* (Onishi et al., 2020). In addition, most in vivo fluorescence studies of *C. reinhardtii* struggle with low temporal or optical resolutions and cover only very short time periods (Lechtreck et al., 2002; Ruiz-Binder et al., 2002; Schoppmeier et al., 2005; Liu et al., 2017). To date, a comprehensive in vivo study of green algae mitosis is clearly lacking.

Although *C. reinhardtii* is usually used to study mitosis in Chlamydomonadales, *V. carteri* is better suited for this purpose, especially because the reproductive cells of *V. carteri* are at least 100 times bigger than those of *C. reinhardtii*, providing a significant size advantage that facilitates analysis of subcellular structures by fluorescence microscopy.

In this study, we generated the required conditions for live-cell imaging of cell divisions in *V. carteri*. Promising candidate proteins for fluorescent labeling were first identified by in silico data analysis and the corresponding genes were then cloned and fused to the *yfp* gene. After generating stable transgenic *V. carteri* strains expressing these genes, we finally analyzed six different proteins during interphase and embryonic cell divisions of *V. carteri* by live-cell imaging. We find enormous dynamics of the microtubule cytoskeleton during centrosome separation, spindle formation, the establishment of the phycoplast, and the generation of previously unknown microtubule-based structures. We show that the centrosomes participate in initiation of spindle formation and define the orientation of the spindle. Even without nuclear envelope breakdown during early mitosis, we demonstrate a temporary loss of nuclear identity. We finally present a model that describes structural and temporal relationships during mitosis in *V. carteri*.

## Results

### Generation of the required conditions for live-cell imaging of cell divisions in *V. carteri*

To visualize the molecular and cellular dynamics during embryonic cell division of *V. carteri*, we searched for mitosis and cytokinesis-associated proteins with known subcellular localization in other organisms and identified the homologous proteins and their genes in *V. carteri*. Details of the in silico selection procedure can be found in the “Materials and methods.” We finally used histone H2B, β-tubulin TubB2, Ran GTPase activating protein 1 (RanGAP1), and dynamin-related protein 1 (DRP1). To produce gene fusions with *yfp*, the corresponding DNA fragments were cloned, assembled, and sequenced. In addition to these four fusions, we connected *yfp* with a sequence encoding only a nuclear localization signal (NLS) and we produced a variant entirely without protein targeting signal. The six final vectors are shown schematically in Supplemental Figure S1. For each vector, we then generated multiple independent, stable transgenic *V. carteri* strains producing the corresponding fluorescent proteins. Transformants used for further analyses show the wild-type phenotype (Supplemental Figure S2). Likewise, the measured indicators of growth in transformants correspond to those of wild-type algae (Supplemental Figure S3). We also compared the expression levels of the genes in question in the wild-type with those in the respective transformants (Supplemental Figure S4). The results show that expression of the additional gene fusions with *yfp* leads to only a moderate increase in the overall expression of the corresponding genes in the transformants. The fluorescence signal of the YFP marker could be unambiguously identified in the respective transformants by spectral analysis using the lambda scan function of the confocal microscope. Each of the six proteins always resulted in the same typical distribution of fluorescence in independent transformed strains. For all protein localizations described below, Supplemental Data Set S1 provides an overview of replications for each observed structure, process, and topology using a specific fluorescent protein. In addition, Supplemental Table S1 shows an overview of replications in terms of coverage of the mitotic phases separately for each fluorescent protein used.

### YFP:NLS reveals characteristics of cell nuclei during early embryonic cell divisions

**Figure 2:**
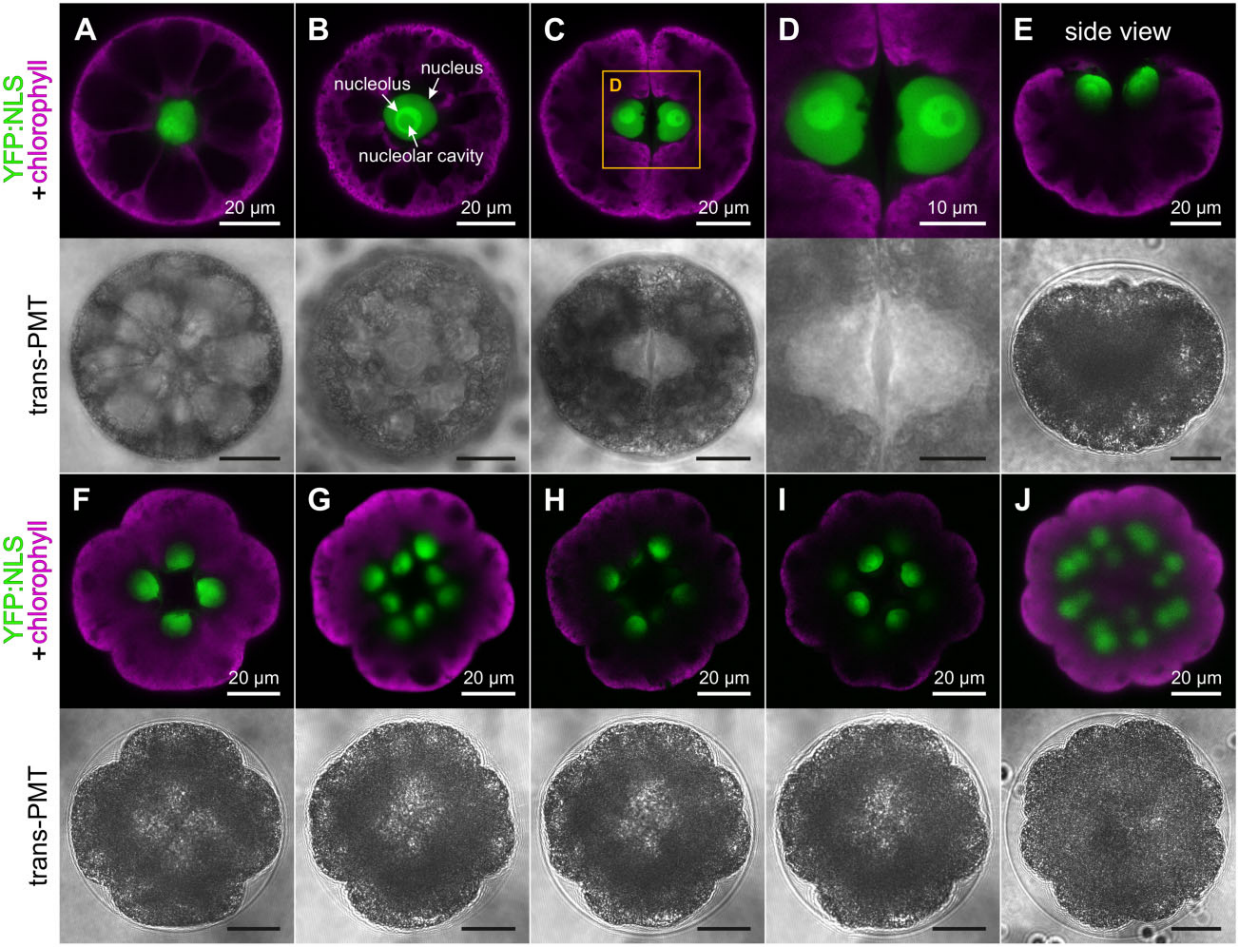
Positioning of cell nuclei during the early embryonic cell divisions visualized by YFP:NLS. In vivo CLSM imaging of *Volvox* transformants producing the nuclear localized YFP:NLS (green). The chlorophyll fluorescence (magenta) is shown for orientation. The corresponding transmission-PMT (trans-PMT) images are below the fluorescence images. With the exception of the side view in E, all images are top views onto the anterior poles of gonidia or embryos. A, Interphase gonidium before the first cell division; the focal plane approximately bisects the cell showing that the nucleus is in the center of the cell. B, Embryo undergoing the first mitotic division. The focal plane is slightly below the surface of the cell as the nucleus migrated closely to the surface of the cell. C, Two-celled embryo with flattened interphase nuclei. D, Enlarged view of the framed section in C. E, Side view of a two-celled embryo with nuclei located at the anterior poles of the cells. F, Four-celled embryo with four interphase nuclei. G–I, Three optical sections of an early eight-celled embryo: G is a section of 3.2 µm showing all nuclei of the embryo; H is a 0.8-µm section showing only the four upper nuclei, and I is a 0.8-µm section showing only the four subjacent nuclei. The distance between the upper and lower plane of nuclei is approximately 6 µm. J, Transition from the 8-cell to the 16-cell stage. All nuclei have already divided whereas the contour of the embryo still looks like that of an 8-celled embryo.

*Volvox* *carteri* transformants expressing *yfp* fused to a NLS (Supplemental Figure S1A) were examined by confocal laser scanning microscopy (CLSM). In these transformants, YFP:NLS protein is clearly imported into the nucleus. During interphase, YFP:NLS accumulates strongly in the nucleoplasm and even more in the nucleolus (Figure 2, A–J). Before initiation of embryonic cell divisions, the nucleus is in the center of the gonidium, so the cellular structures above the nucleus somewhat quench and scatter the YFP fluorescence (Figure 2A). At the beginning of embryonic cell divisions, the anterior pole of the gonidium (i.e. the pole that faces the external surface of the spheroid) flattens. This change in shape is accompanied by an approach of the nucleus to the anterior surface of the gonidium (Figure 1C), which causes the chloroplast and vacuoles to be displaced laterally, resulting in a clear top view onto the nuclear structure (Figure 2B). The nucleoli are almost perfectly round and have irregular dark spots inside, the nucleolar cavities, which change dynamically in shape, size, and number.

The first embryonic division of the gonidium is symmetrical and in the resulting two-celled embryo the two nuclei lie directly next to each other. Both the cells and the nuclei are flattened on the side where they face each other (Figure 2C) and the surface of the flattened side of the nuclear envelope frequently is uneven (Figure 2D). During the first two embryonic division cycles, the nuclei of the cells are always close to the anterior cell surfaces (Figure 2E). From the second division cycle onward, the division planes show an increasingly oblique position relative to the anterior–posterior axis. In the further course of development, the nuclei move to the inner surface of the embryonic hollow sphere, becoming increasingly obscured by the chloroplasts, which attenuates and blurs the YFP fluorescence (Figure 2J).

**Figure 3:**
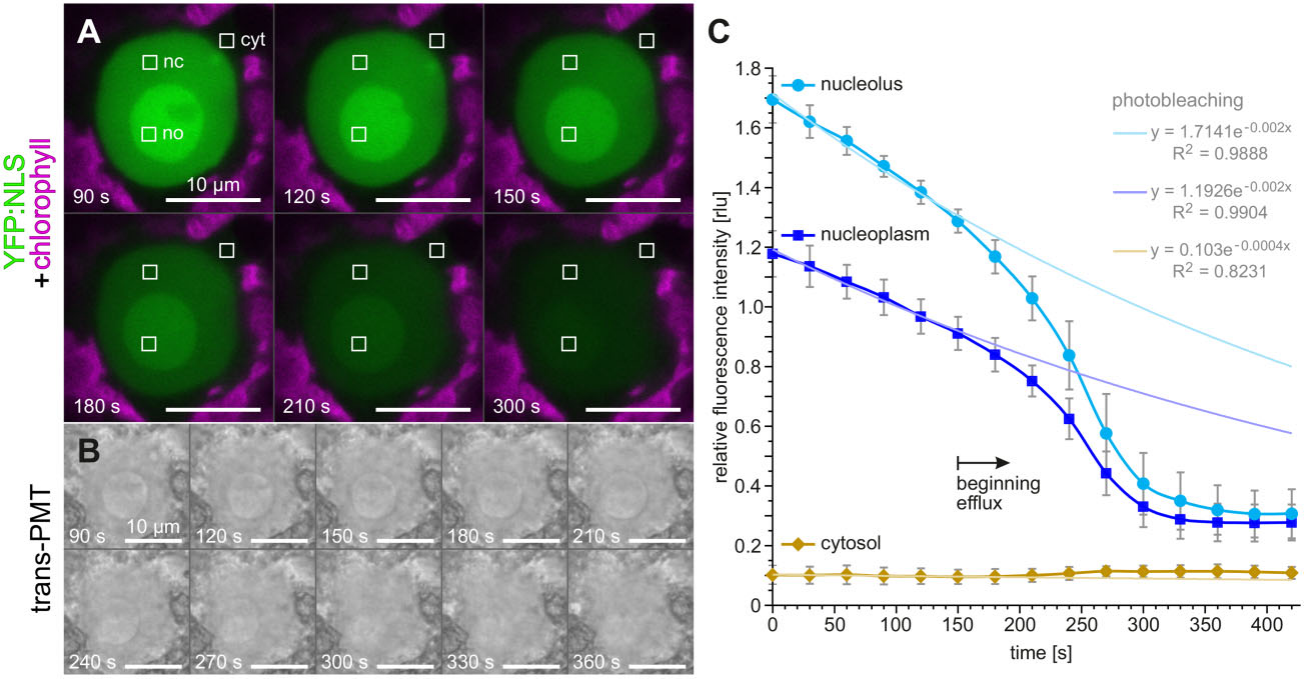
Efflux dynamics of nuclear YFP:NLS at prophase. In vivo CLSM analysis of the first embryonic cell division using *Volvox* transformants producing YFP:NLS. Time-series analysis of the efflux of nuclear YFP:NLS during disintegration of the nucleolus in prophase. A and B, Top view onto the nucleus of a dividing gonidium. The time specifications in the lower left corners correspond to the time specifications in C. A, CLSM imaging of YFP:NLS (green). The chlorophyll fluorescence (magenta) is shown for orientation. Exemplary regions of interest (ROIs) used for determination of fluorescence intensity over time are marked (white squares) in the cytosol (cyt), nucleoplasm (nc), and nucleolus (no). B, Transmission-PMT images taken simultaneously with the time series shown in A. C, Time series plot showing changes in distribution of YFP:NLS fluorescence over time. For four different gonidia, square ROIs were selected as exemplified in A. The mean intensity of the selected ROIs was determined for each time point and normalized by the half-maximum fluorescence intensity of the nucleolus. Error bars indicate the standard deviation. The beginning of the YFP:NLS efflux is marked. The decrease of fluorescence intensity due to photobleaching was approximated by exponential regression lines using the first six points in time.

As soon as the gonidia approach the first embryonic cell division, YFP:NLS translocates from the nuclear structures to the cytosol (Figure 3A and Supplemental Movie S1) and simultaneously the nucleolus disintegrates (Figure 3B). A time series plot shows that the decrease of nuclear and nucleolar fluorescence is clearly greater than the signal loss due to photobleaching (Figure 3C). YFP:NLS reaches its final distribution between the cytosol and nucleoplasm within only about 5 min after the onset of efflux into the cytosol. Why the fluorescence in the cytosol then increases only from 0.093 to 0.11 rlu can be explained by the fact that the volume of the cytoplasm exceeds the volume of the nucleus by at least 30 times. Even though the YFP:NLS fluorescence measured in the nucleus is about 2.5 times higher than that in the cytosol, it can be assumed that the actual concentrations of the fluorescent protein in the nucleoplasm and cytosol are approximately the same due to influences from other cell components, as shown in Supplemental Figure S5. These findings suggest that the nuclear envelope is no longer a barrier at this stage of development. In telophase, when nuclear envelope function including nuclear transport is restored, YFP:NLS is again strongly concentrated in the nucleus. To show the overall efflux and influx dynamics during the first cell division, we tracked individual cells from prophase to cytokinesis and measured YFP:NLS fluorescence continuously (Supplemental Figure S6 and Supplemental Movie S2). The efflux from the nucleus is about five times faster than the later influx into the new nuclei, which takes approximately 25 min (Supplemental Figure S6). After correcting for photobleaching, it is clear that the nuclear signal intensity returns to approximately the same level as before division (Supplemental Figure S6).

Not only does YFP:NLS leak from the nucleus during the first cell division, but also during the following divisions. The fluorescence distribution in both subsequent prophases and subsequent metaphases is similar to that in the first prophase and first metaphase, respectively (Supplemental Figure S7). After the fourth cell division, it is no longer possible to make a statement about this because the fluorescence signal becomes increasingly obscured by the chloroplasts.

### Cytosolic and nucleoplasmic processes during mitosis illuminated by pts-free YFP

**Figure 4:**
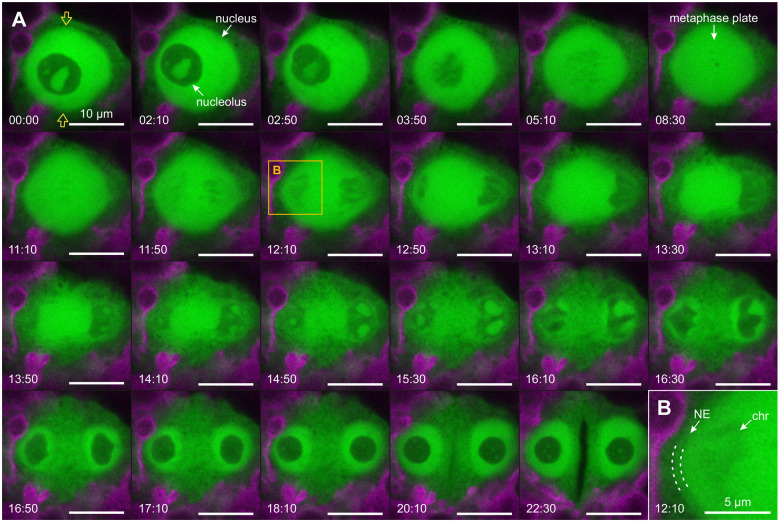
Visualization of the first embryonic mitosis by pts-free YFP. A, In vivo CLSM-time series for imaging of the first embryonic division using *Volvox* transformants that produce YFP without any protein targeting signal (green). The chlorophyll fluorescence of chloroplasts (magenta) is shown for orientation. Top view onto the nucleus of a dividing gonidium. The series starts at late prophase and ends with the formation of the cleavage furrow in the nuclear plane during cytokinesis. B, Enlarged view of the framed section in A showing the location of the nuclear envelope (NE) and a uniformly stained, approximately 0.5-µm-thick layer (dashed lines) that surrounds the nucleus. chr, chromatids. The time difference in relation to the first image is given in min:s.

In *V. carteri* transformants that produce YFP without any protein targeting signal (pts-free YFP) (Supplemental Figure S1B), fluorescence signals are clearly detectable during all phases of the mitotic cycle not only in the cytosol but also in the nucleus (Figure 4 and Movie 1) because the small size of pts-free YFP allows its diffusion through the nuclear pores (Wei et al., 2003; Seibel et al., 2007). The fact that the cell nucleus even appears much brighter than the cytosol is due to influences of other cell components (such as membrane-enclosed organelles), as shown in Supplemental Figure S5. When these influences are taken into account, the concentration of fluorescent proteins in the nucleoplasm and cytosol becomes comparable. At the beginning of mitosis, the gonidial nucleolus has an average diameter of 7.74 µm (SD 0.47 µm) and the diameter of the nucleus is on average 15.39 µm (SD 0.81 µm), which means that the nucleolus occupies roughly an eighth (more precisely 12.95%, SD 2.74%) of the nuclear volume (Figure 4A and Supplemental Data Set S2). In prophase, the nucleolus moves close to the nuclear envelope. The outline of the nucleolus becomes increasingly irregular and blurry and, eventually, the nucleolus disintegrates completely (Figure 4A, first row). In contrast, the nucleus retains its shape during prophase and metaphase. The condensed chromosomes are silhouetted against the uniformly stained nucleoplasm and can be most easily identified as such in anaphase when the two pairs of sister chromatids separate from each other (Figure 4A at 11:10 to 12:50). During anaphase, the nucleus elongates perpendicular to the division plane. Simultaneously, the chromatids lose their contours, which indicates their decondensation. Immediately after the chromatids have reached the spindle poles, an extensive restructuring of the nuclear envelope begins (Figure 4A at 12:50 to 13:30). The previously strongly fluorescent nucleus dissolves more and more and this progresses from outside to inside. Slightly darker structures, which are presumably membrane sheets, emerge first in the uniformly stained nucleoplasm at the edge of the nuclear division plane (Figure 4A at 13:30 to 14:50). Simultaneously, the nuclear envelopes of the daughter cells begin to form around the two decondensing sister chromatid sets. Portions of the negatively stained chromatids are directly transformed into the two new nucleoli, which gradually detach from the newly formed nuclear envelopes. The staining with pts-free YFP also reveals two layers surrounding the nucleus: A thin dark layer (≤20 nm), which is difficult to discern and corresponds to the nuclear envelope, and, directly above, an approximately 0.5 µm thick, uniformly stained layer, which stands out from the surrounding cytoplasm (Figure 4B).

### Structure and dynamics of chromatin during the first embryonic cell division

**Figure 5:**
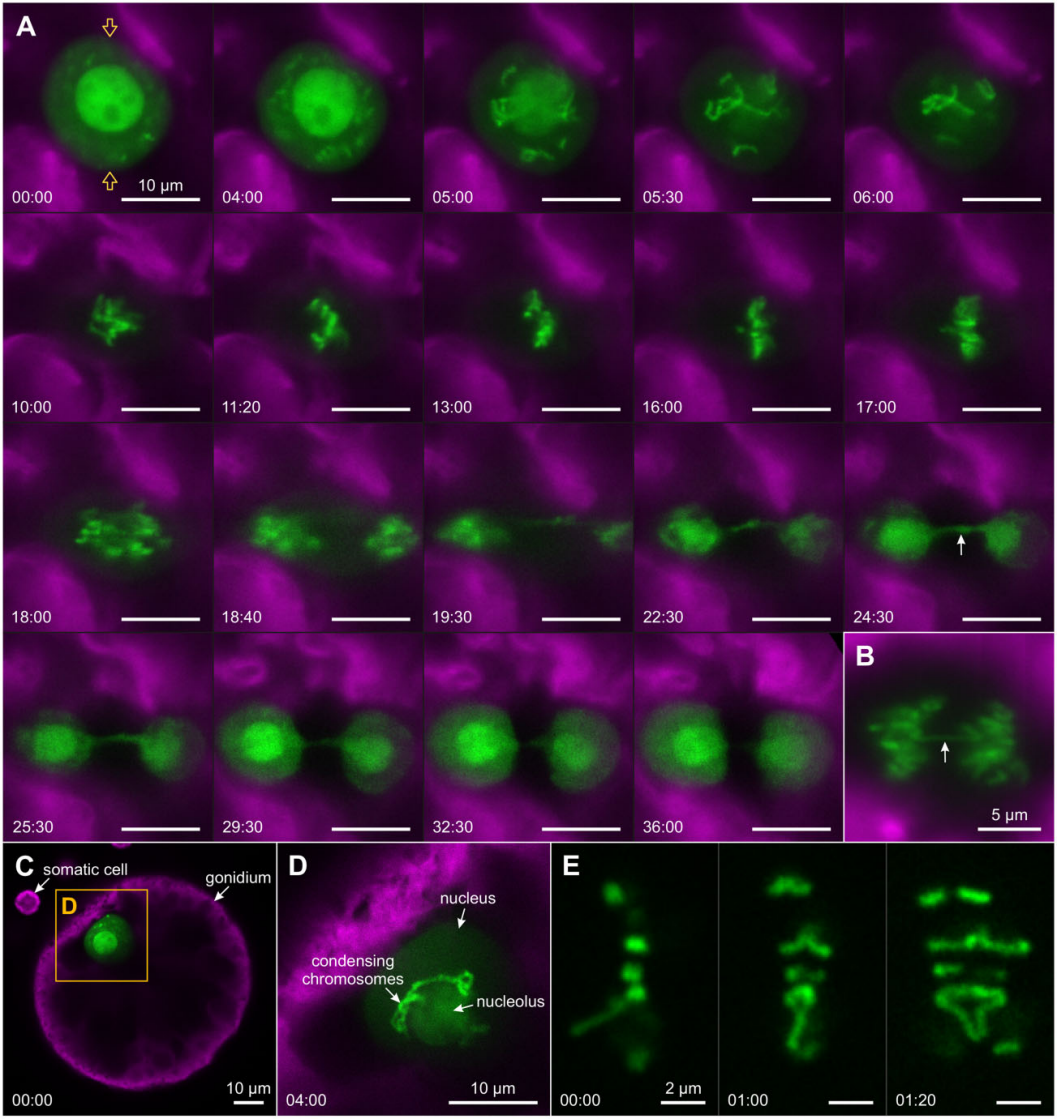
In vivo tracking of chromatin with H2B:YFP during the first embryonic cell division. In vivo CLSM imaging of chromatin in gonidial nuclei using *Volvox* transformants that produce fluorescent histone H2B:YFP (green). The chlorophyll fluorescence of chloroplasts (magenta) is shown for orientation. A, Image series starting at late prophase and ending with the complete separation of the two daughter nuclei during cytokinesis. Two orange arrows indicate the position of the division plane. A prolonged chromatid cohesion leads to formation of an anaphase bridge (white arrow at 24:30). B, Detailed view of two separating sets of chromatids with a filamentous connection, the anaphase bridge (arrow). C, Oblique view of a dividing gonidium at late prophase with its nucleus located at the anterior pole of the cell. D, Enlarged view of the framed section in C 4 min after C was captured showing condensing chromosomes. E, Image series of separating sister chromatids during anaphase. The images in A, B, and E are top views onto nuclei at the anterior poles of dividing gonidia, whereas C and D show oblique views. Time differences in relation to the first image of an image series are given in min:s.

The phases of the mitotic cycle are defined primarily by the degree of condensation of chromatin, making chromatin visualization essential for analysis of mitotic processes. In transformants that express the *V. carteri h2b* coding sequence fused to *yfp* (Supplemental Figure S1C), YFP-tagged histone H2B allowed us to localize chromatin and, consequently, chromatids and chromosomes in living cells (Figure 5 and Movie 2). Before mitosis, H2B:YFP is distributed throughout the nucleoplasm and particularly accumulates in the nucleolus. At late prophase, patches of H2B:YFP in the nucleoplasm outside the nucleolus indicate the presence of condensed chromatin (Figure 5A at 00:00). The accumulation and further condensation of chromosomes occurs mainly in the vicinity of the nucleolus and simultaneously with the disintegration of the nucleolus (Figure 5A at 04:00 to 10:00, Figure 5, C and D). While the chromosomes condense, they become progressively brighter (Figure 5A at 04:00 to 13:00), not only because of the increasing packing density, but also because strong condensation reduces the dissociation rate of histone H2B:YFP, stabilizing its chromatin binding (Martin and Cardoso, 2010). The chromosomes then congress at the metaphase plate and once all chromosomes are gathered there, the transition to anaphase is initiated (Figure 5A at 06:00 to 16:00). The sister chromatids then gradually lose their cohesion to each other and are drawn to the respective spindle pole. The separation of the sister chromatids starts at the centromeres and progresses from there to the ends of the chromosomes (Figure 5E). Quite frequently, prolonged cohesion between two chromatids leads to the formation of anaphase bridges (Figure 5A at 24:30), which usually dissolve during late anaphase or telophase. During anaphase, chromatids begin to decondense (Figure 5A at 18:40), with portions of the chromatids being converted directly into the two new nucleoli (Figure 5A at 18:40 to 29:30).

### Substantial changes of the microtubular cytoskeleton during early mitosis

**Figure 6:**
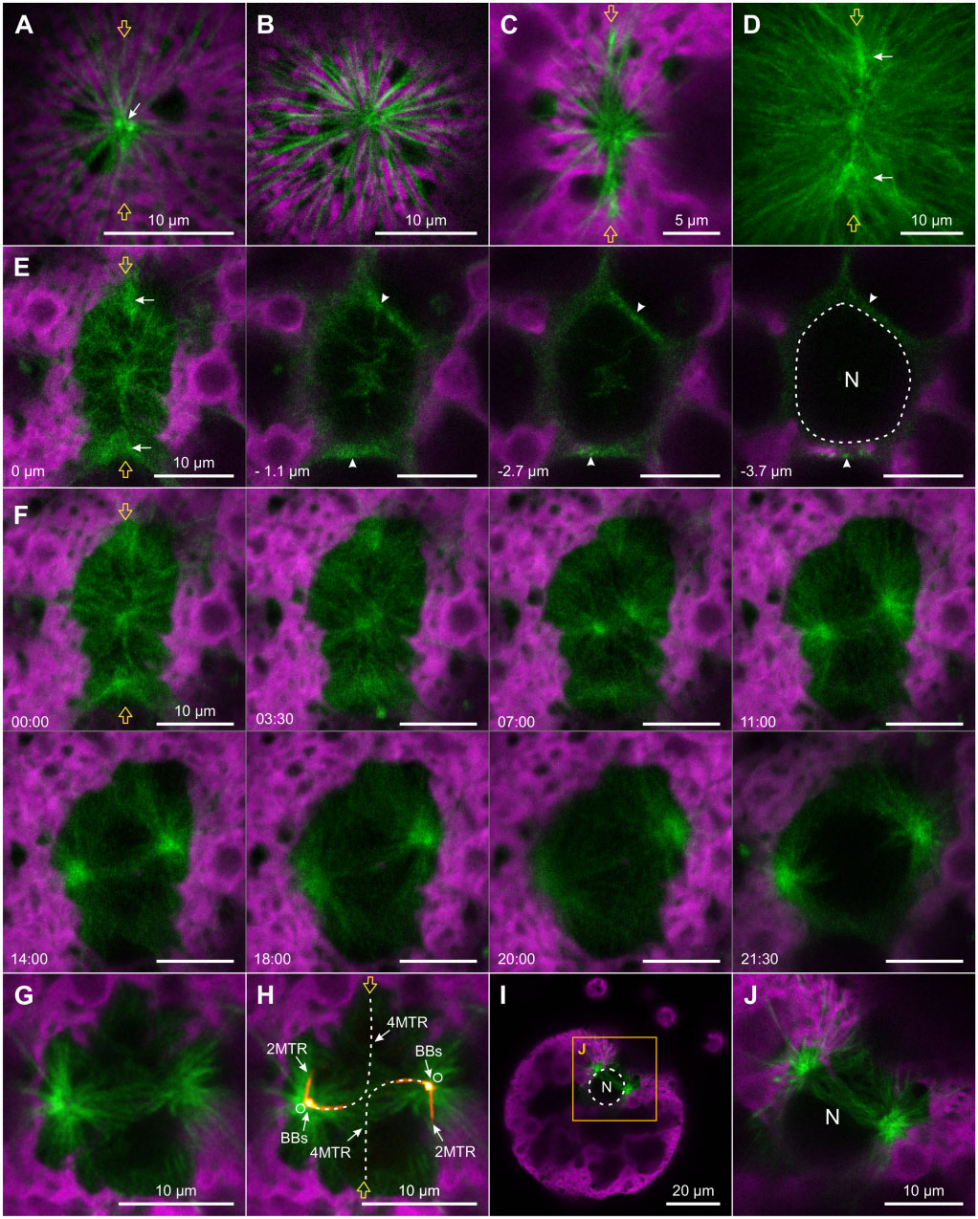
MTOC division and early changes of microtubular structures visualized by YFP:TubB2. In vivo CLSM imaging of microtubular structures before and during prophase of the first embryonic cell division using *Volvox* transformants that produce fluorescent YFP:TubB2 (green). The chlorophyll fluorescence of chloroplasts (magenta) is shown for orientation. Two orange arrows indicate the position of the division plane when appropriate. A, Microtubular structures of an interphase gonidium. Cytoplasmic microtubules emerge near the two basal bodies (white arrow), which are located directly below the surface of the cell. The image section shows only the proximal part of the very long cytoplasmic microtubules. B–J, Reorganization of the microtubule cytoskeleton during preprophase and prophase. B, In preparation for mitosis, cytoplasmic microtubules reorganize into microtubule bundles. C, Shortly afterwards, the bundles become more concentrated along the division plane. D, Then, two additional centers of YFP:TubB2 fluorescence (white arrows) form on the division plane. Maximum intensity projection of the microtubule cytoskeleton. E, *Z*-stack images of the microtubule cytoskeleton just before spatial separation of the two halves of the duplicated centrosome. Both the microtubules of the centrosome and those of the two additional centers of fluorescence (white arrows) reach into the cell (arrowheads) and enclose the nucleus (N, dashed line). The spatial depth of each optical slice is indicated in comparison to the first slice. F, Time-series images showing the spatial separation of the two halves of the duplicated centrosome during prophase. For taking the image at 21:30, the focal plane had to be adjusted because the centrosomes gradually sank ever deeper into the cell. Time differences in relation to the first image are given in min:s. G, Detailed view of the microtubular structures during centrosome separation. H, Same as in G but with additional indication of the position of basal bodies/centrioles and microtubular rootlets. The older basal body/centriole and the proximal parts of the rootlet microtubules are located by overlaying an image that resolves the localization of YFP-tagged basal body protein Babo1 (Babo1:YFP in orange hot) (von der Heyde and Hallmann, 2020). The position of younger basal bodies/centrioles (empty circles) and four-membered rootlet microtubules (dashed lines) was estimated based on earlier publications (Holmes and Dutcher, 1989; Kirk et al., 1991; Ehler et al., 1995). BBs, basal bodies/centrioles; 2MTRs, two-membered microtubular rootlets; 4MTRs, four-membered microtubular rootlets. I, Oblique view of a gonidium during centrosome separation. The approximate contour of the nucleus (N) is indicated (dashed line). J, Enlarged view of the framed section in I. The images in A–H are top views onto the anterior pole of dividing gonidia, whereas I and J show oblique views.

YFP-tagged tubulin TubB2 (Supplemental Figure S1D) allows the subcellular localization of three different microtubular structures involved in microalgal cell division: centrosomal asters, the mitotic spindle, and the phycoplast. During interphase, the basal apparatus is located immediately adjacent to the gonidial cell membrane, at the point closest to the outer surface of the organism (Figure 1B). The basal apparatus, which in *Volvox* is also the centrosome, contains two mature basal bodies/centrioles and serves as a central MTOC, nucleating numerous microtubules that extend throughout the cell (Figure 6A). In interphase, the distance between the two basal bodies/centrioles is only 0.84 µm (SD 0.09 µm) (Supplemental Data Set S2). One of the first signs of the onset of mitosis is the shortening of the long cytoplasmic microtubules and their reorganization into microtubule bundles. These microtubule bundles are radially organized around the basal apparatus/centrosome (Figure 6B). This development coincides with the elongation of the two procentrioles (nascent centrioles), whereupon the centrosome eventually contains two pairs of mature centrioles, each pair with one newly formed and one preexisting centriole (Gould, 1975; O’Toole and Dutcher, 2014). Subsequently, the radially symmetric shape of the microtubular cytoskeleton changes to an elongated, bilaterally symmetric shape (Figure 6C). The microtubule bundles are more concentrated at the position of the four-membered microtubular rootlets marking the orientation of the division plane (Holmes and Dutcher, 1989; Ehler and Dutcher, 1998). Thus, this elongated, bilaterally symmetric structure exhibits similar positioning and temporal occurrence as the preprophase band of vascular plants. The formation of the bilaterally symmetric microtubule arrangement is accompanied by an initial furrowing of the anterior part of the chloroplast. The nucleus and cell membrane approach each other and two additional centers of YFP:TubB2 fluorescence are formed in the division plane (Figure 6D). The additional centers of fluorescence are located just above the nuclear surface and they form microtubule bundles that extend toward and partially enclose the nucleus (Figure 6E). Subsequently, the centrosome divides and the two resulting centrosomes move away from each other (Figure 6F). The imaginary line between the two separating centrosomes is at an average angle of 68.55° (SD 3.55°) to the imaginary line between the previously formed additional centers of fluorescence (with the centrosome in between) (Supplemental Data Set S2); that is the lines are not perpendicular to each other. During the separation of the centrosomes, they mature, nucleate an increasing number of microtubules, and develop pronounced microtubule asters (Figure 6F). At the same time, the cytoplasmic microtubules, which form the additional centers of fluorescence and which surround the nucleus, dissolve (Figure 6F). The distance between the two centrosomes increases at an average rate of 0.89 µm per minute (SD 0.18 µm/min) (Supplemental Data Set S2) and the centrosomes follow the curvature of the nuclear envelope. Numerous bundles of microtubules are detectable between the two separating centrosomes (Figure 6G). Among the microtubule fibers between the MTOCs are also the microtubular rootlets that connect the two pairs of centrioles (Figure 6H).

**Figure 7:**
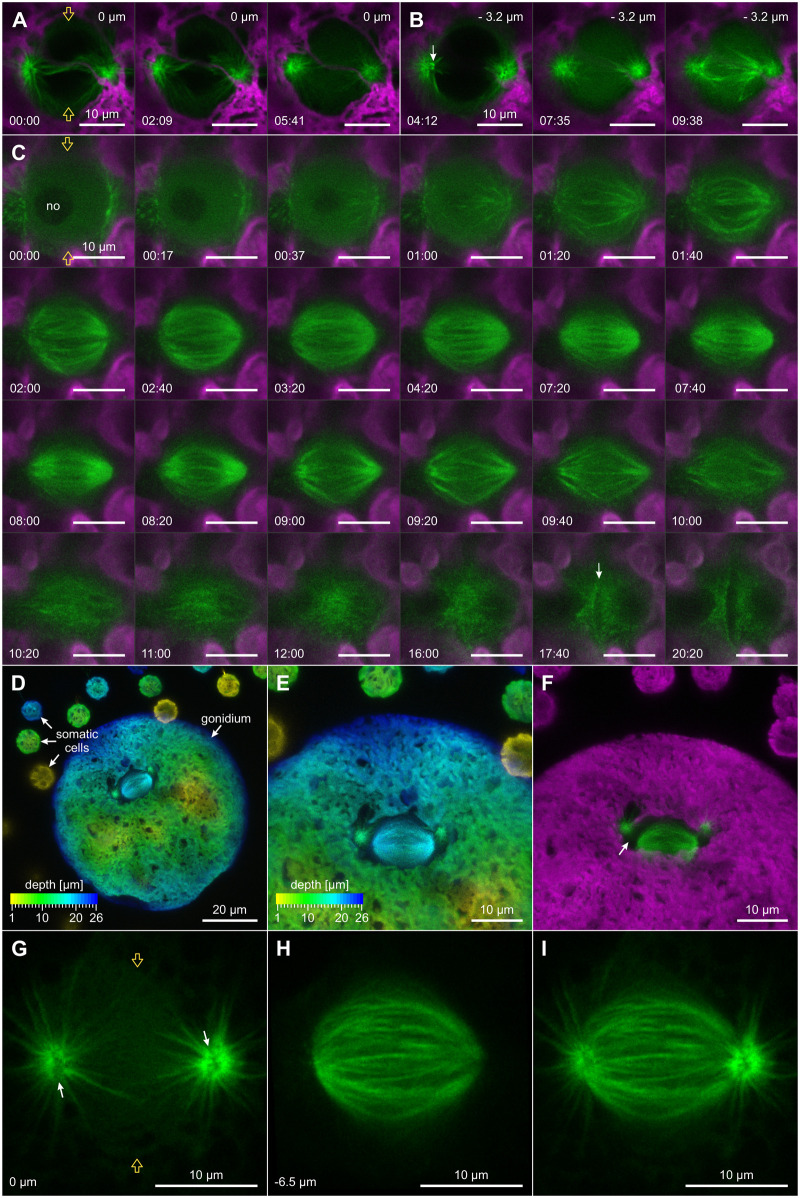
Structural and topological dynamics of the microtubule asters and the spindle apparatus. In vivo CLSM imaging of the first embryonic division of *Volvox* transformants producing YFP:TubB2. With the exception of the oblique views in D–F, all images are top views onto gonidial nuclei. Two orange arrows indicate the position of the division plane. With the exception of D and E, YFP:TubB2 fluorescence is displayed in green and chlorophyll fluorescence of chloroplasts is shown in magenta. A–C, Time series of the microtubule cytoskeleton from late prophase to cytokinesis. The time difference in relation to the first image is given in min:s. A and B, Selected time-series images from two different focal planes showing translocation of tubulin into the nucleus during early mitosis. The focal plane in A is 3.2 µm above the focal plane in B and both optical sections show parts of the anterior hemisphere of the nucleus. During the given period, the number of connecting microtubules between the centrosomes is reduced (above the nucleus), tubulin enters the nucleus, and then spindle formation inside the nucleus is initiated at the centrosomes. A bright structure extends into the nucleus (white arrow at 04:12). C, Dynamics of the microtubule cytoskeleton from disintegration of the **Figure 7: (continued)** nucleolus (no) during prometaphase to the formation of the cleavage furrow (white arrow) in the nuclear plane during cytokinesis. The images of C were gamma adjusted with a value of 0.7 to improve the overall visibility of microtubular structures. D–I, 3D topology of microtubule asters and spindle apparatus after their detachment. D–F, Maximum intensity projection of *z*-stack images with visualized microtubule asters, spindle apparatus, and chloroplasts. Between the spindle apparatus and the microtubule asters, there is an area without microtubules (white arrow in F). The step size in vertical direction is 1 µm. D and E, Color-coded designation of spatial depth. D, Overview. E, Enlarged view of D. F, Tilted view on the image stack shown in E. G and H, Microtubule asters and spindle apparatus at metaphase. G, Each microtubule aster exhibits a darker stripe through its center (white arrows). H, Spindle apparatus at a focal plane 6.5 µm below the focal plane in G. I, Maximum intensity projection of the layers shown in G and H.

At this developmental stage, as during interphase, YFP:TubB2 was detected exclusively in the cytosol. A side view of the separating centrosomes shows that the connecting microtubule fibers form a straight direct link between the centrosomes, constricting the nuclear envelope and causing an imprint in the unstained and otherwise spherical nucleus (Figure 6, I and J and Supplemental Figure S5). This distinct imprinting of the anterior nuclear envelope is only visible for a short period of time because the microtubular connection between the centrosomes is degraded once the centrosomes have reached their final position, which is approximately where the future spindle poles will be located (Figure 7A). The short-term and one-time occurrence of the imprinting on the nuclear surface makes it a reliable, specific identifier for late prophase and imminent spindle formation for researchers experienced in the field. The imprinting is also visible if, instead of tubulin, components of the nucleoplasm or the cytosol are fluorescently labeled (Supplemental Figure S5), which also allows prediction of spindle orientation. Once at their final position for spindle formation, the distance between centrosomes is 17.47 µm (SD 0.56 µm) (Supplemental Data Set S2).

### Genesis, positioning, and shape of the mitotic spindle

**Figure 8:**
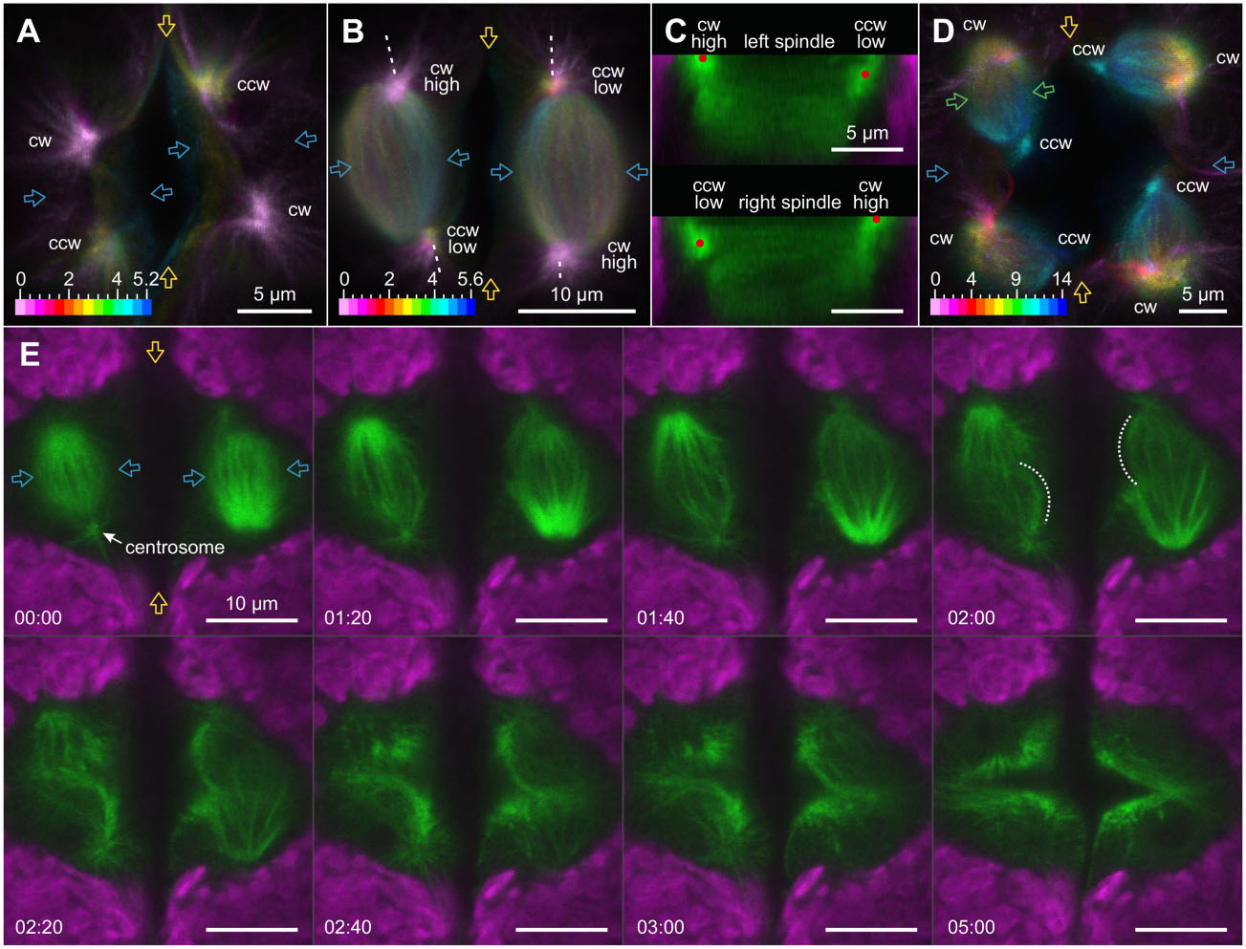
Topology of microtubule asters, spindles, and phycoplasts during the second and third embryonic cell division. In vivo CLSM imaging using *Volvox* transformants that produce YFP:TubB2. Top view onto the microtubular structures of dividing embryos. Clockwise (cw) and counterclockwise (ccw) centrosomes are indicated. A, B, and D, Maximum intensity projection of *z*-stack images with color-coded designation of spatial depth. The depth of the focal planes is given in µm. B and C, “High” means microtubule aster closer to the viewer, “low” means microtubule aster further away from the viewer; see also Supplemental Figure S8. C and E, YFP:TubB2 is shown in green and chlorophyll fluorescence of chloroplasts in magenta. A, Spatial arrangement of the four microtubule asters during prophase of the second embryonic cell division. The step size in vertical direction is 0.4 µm. B, Spatial arrangement of asters and spindles during metaphase of the second mitosis. The step size in vertical direction is 0.4 µm. Dashed lines indicate the cutting lines for the vertical slices shown in C. C, Vertical slices through the spindles of the image stack shown in B illustrating spindle shape and orientation in side view. Red dots indicate the position of the centrosomes. D, Microtubule asters and spindles during the third metaphase. Arrows indicate the approximate position of the first (orange), second (blue), and third (green) division plane. The step size in vertical direction is 1 µm. E, Time-series images of microtubular structures during the second cell division. The series starts at metaphase, ends with the initiation of the cleavage furrow during cytokinesis, and shows the reorganization of spindle tubulin into microtubular structures of the phycoplast. Dotted lines mark some of the emerging hook-like structures of the phycoplast. Time is in min:s.

Once the separation of the centrosomes is complete, both centrosomes are in close proximity to the nucleus (Figure 7A). The sides of the microtubule asters facing the nucleus are flattened and occasionally there are areas of intense YFP:TubB2 fluorescence extending into the nucleus (white arrow in Figure 7B at 04:12). Once free tubulin enters the nucleus, the microtubule asters become radially symmetric (Figure 7A at 05:41) and shortly thereafter, spindle microtubules begin to emerge near the centrosomes (Figure 7B at 07:35). Time-series images in Figure 7C document the complete development of the spindle structure (see also Supplemental Movie S3 and Movie 3). Note that in these images the focal plane bisects the spindle, which is why the overlying microtubule asters are not visible. The disintegration of the nucleolus begins approximately at the start of spindle formation (Figure 7C, first row). The formation of the spindle is accompanied by an attenuation of the YFP:TubB2 fluorescence signal of microtubule asters indicating a relocation of any available tubulin to spindle microtubules. Shortly after induction of spindle formation, the MTOCs move away from the spindle poles. In metaphase, the length of the spindle is then on average 14.83 µm (SD 0.74 µm) (Supplemental Data Set S2). Maximum intensity projections of vertical stacks illustrate the precise topology of the microtubule asters and the spindle inside the gonidium (Figure 7, D–I). The topology becomes particularly clear in a 3D projection (Supplemental Movie S4). Dividing gonidia are slightly flattened and have a central pit on their anterior surface. The spindle apparatus with the overlying microtubule asters is located just below the central pit. During mitosis, the connection of spindle poles and microtubule asters is loosened and a distinct gap is formed between the two microtubular structures (white arrow in Figure 7F and Supplemental Movie S4). The vertical distance between the centrosomes and the corresponding spindle poles is about 5 µm (mean 5.02 µm, SD 0.39 µm) (Supplemental Data Set S2). The centrosomes are located directly above the spindle poles (Figure 7, G–I) and there is an irregular dark stripe through their center (white arrows in Figure 7G). During the centrosome separation of the second embryonic mitosis, the two centrosomes that are positioned relative to their corresponding sister centrosomes in clockwise direction (as seen from above), the clockwise centrosomes, are located closer to the anterior surface of the embryo, but farther from the first cleavage furrow than the counterclockwise centrosomes (Figure 8A and Supplemental Figure S8). This spatial arrangement is also retained during metaphase (Figure 8, B and C and Supplemental Figure S8). The position and oblique orientation of the two spindles are obviously determined during the separation of the corresponding centrosomes and they correspond to the relative positions of the resulting daughter cells to each other (Figures 8D and 2, G–I and Supplemental Figure S8). In top view, the *Volvox* spindle changes from a round to an oval shape during metaphase (Figure 7C, second row). The central part of the spindle then becomes darker than the poles, probably due to quenching of the YFP:TubB2 fluorescence by the accumulation of chromosomes at the metaphase plate. During anaphase, the mitotic spindle elongates with an average speed of 3.34 µm/min (SD 0.35 µm/min) to a maximum length of 21.31 µm (SD 1.51 µm), corresponding to an average length increase of 43.72% (SD 6.6%) (Supplemental Data Set S2). At the same time, the spindle microtubules are reduced to a few but more pronounced microtubule bundles. The diamond shape of the spindle indicates that the polar microtubules no longer interdigitate at the spindle midzone with polar microtubules from the opposite pole and thus their connection has been lost (Figure 7C at 09:20). Once the chromatids reach the spindle poles, the spindle disintegrates completely (Figure 7C at 10:00 to 12:00). The time between the first signs of spindle elongation and the complete disintegration of the spindle is on average only 130.52 s (SD 10.1 s).

### Formation and structure of the phycoplast

**Figure 9:**
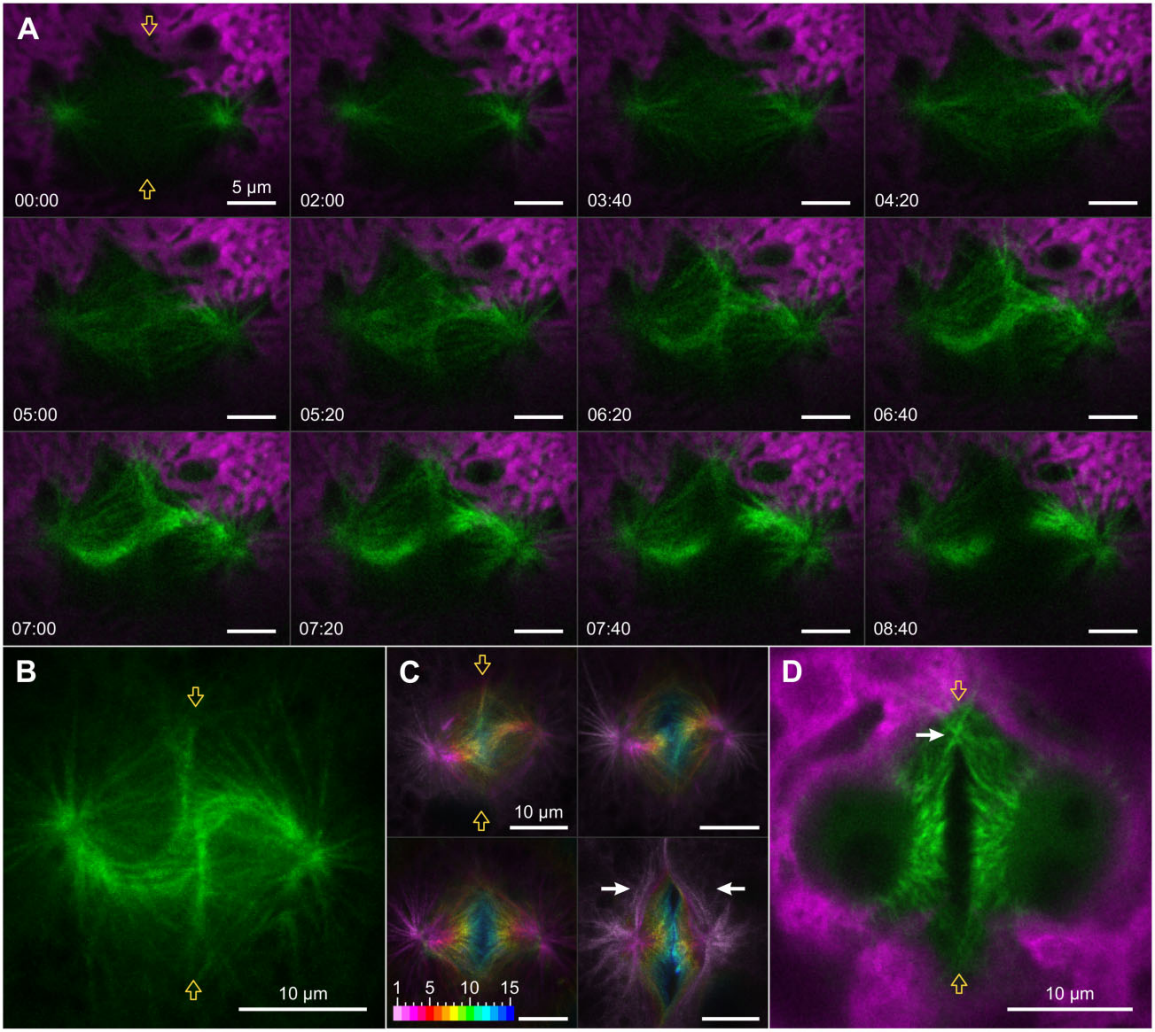
Microtubule-based structures of the phycoplast during the first embryonic cell division. In vivo CLSM imaging using *Volvox* transformants that produce YFP:TubB2. Top view onto the phycoplast. Two orange arrows indicate the position of the division plane. Except for C, YFP:TubB2 is shown in green and chlorophyll fluorescence of chloroplasts in magenta. A, Time-series images showing the concentration of microtubule bundles between the two centrosomes of the dividing gonidium building a microtubule structure that looks like two point-symmetric hooks. The series starts at metaphase and ends with the formation of the cleavage furrow during cytokinesis. Note that from 7:00 onward the middle part of the structure moves downward and leaves the image plane. The time difference in relation to the first image is given in min:s. B, Maximum intensity projection showing the microtubular network of the phycoplast as two point-symmetric hooks. C, Maximum intensity projection of *z*-stack images with color-coded designation of spatial depth showing the microtubular network of the phycoplast at different stages of cytokinesis. The microtubular structures are visualized by YFP:TubB2 fluorescence. The spatial depths of the focal planes are given in µm. The step size in vertical direction is 1 µm. White arrows indicate a decrease in the distance between the centrosomes during formation of the cleavage furrow. D, Advanced stage of cytokinesis showing the microtubular network at the cleavage furrow and a negative display of the two newly formed nuclei. Microtubules of the opposing daughter cells overlap at the edge of the cleavage furrow (white arrow).

Closer examinations of the microtubular structures reveal that phycoplast formation proceeds along the microtubular rootlets (Figure 9A and Supplemental Movie S5). The phycoplast increasingly resembles two point-symmetric hooks, with one end of each hook at a centrosome and the other end on one side of the cell division plane. The detailed structure of the fully formed phycoplast is illustrated by a maximum intensity projection of a vertical image stack (Figure 9B). The phycoplast exhibits 180° rotational symmetry and its curvature reflects the direct involvement of the microtubular rootlets in its formation (compare Figure 9, A and B to Figure 6H). The examination of both the first and second embryonic cell division shows that the total fluorescence of the phycoplast increases while the spindle disintegrates (first division: Supplemental Figure S9 and Movie 3; second division: Figure 8E and Supplemental Movie S6). Tubulin released during spindle degradation could be directly reused for phycoplast construction and strengthening (Movie 3). The strengthening of the phycoplast appears to be immediately followed by the cell membrane invagination of the cleavage furrow at the division plane (Supplemental Figure S10 and Movie 3). In YFP:TubB2 fluorescence staining, this is indicated by the rapid reduction of fluorescence in the center of the image, which is due to the middle section of the phycoplast moving toward the posterior hemisphere of the gonidium (Figure 9A at 07:00 to 08:40). 3D stacks reveal that this movement of the middle section of the phycoplast coincides with the progressing invagination of the cleavage furrow (Figure 9C). The middle section of the phycoplast eventually consists of vertical microtubule bundles that are approximately parallel to the advancing cleavage furrow and perpendicular to the previous axis of the mitotic spindle (Figure 9D). The topology of the phycoplast during early and advanced cytokinesis of the first embryonic cell division becomes particularly clear in 3D projections of image stacks (Supplemental Movies S7 and S8, respectively). Microtubules of the opposite halves of the phycoplast overlap at the edge of the progressing cleavage furrow (first division: white arrow in Figure 9D; second division: Figure 8E at 05:00), suggesting their direct involvement in cytokinesis.

### Relocation of RanGAP1 during the cell cycle

**Figure 10:**
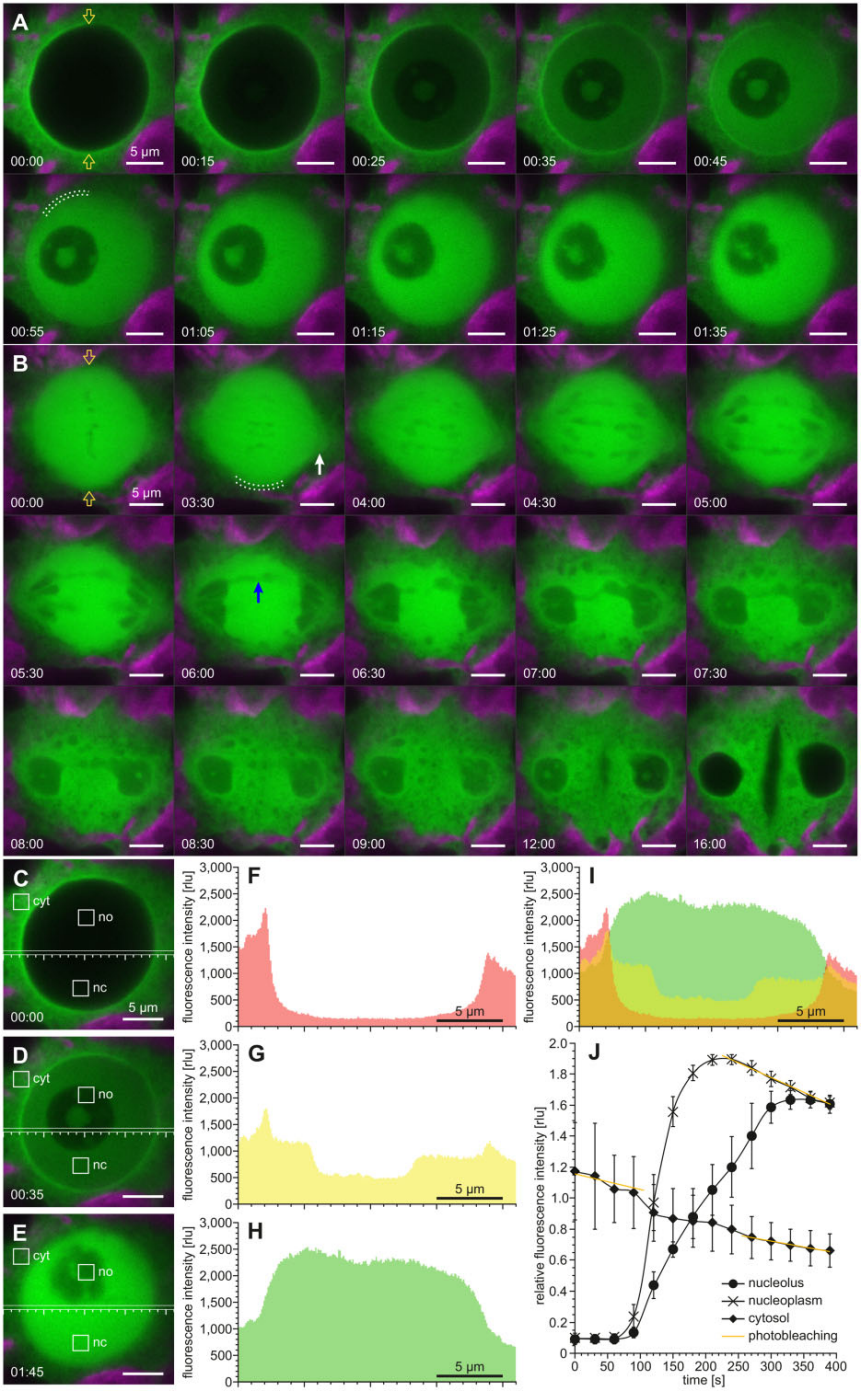
Dynamic localization of RanGAP1:YFP during the first embryonic mitosis. A–E, In vivo CLSM imaging of *Volvox* transformants that produce fluorescent RanGAP1:YFP (green). The chlorophyll fluorescence of chloroplasts (magenta) is shown for orientation. Top view onto a dividing gonidium. Two orange arrows indicate the position of the division plane. The time difference in relation to the first image is given in min:s. A, Time-series images illustrating the influx of RanGAP1 into the nucleus at late prophase and the beginning disintegration of the nucleolus. B, Time-series images of the later RanGAP1 localization starting at late metaphase and ending with the formation of the cleavage furrow during cytokinesis. Note that the nucleus is surrounded by a 0.5-µm-thick continuous layer (dotted lines in A at 00:55 and in B at 03:30), which is much thicker at the spindle poles (white arrow in B at 03:30). Negative staining of chromatids by RanGAP1:YFP shows that a prolonged chromatid cohesion leads to formation of an anaphase bridge (blue arrow in B at 06:00). C–J, Analysis of influx dynamics of cytosolic RanGAP1:YFP into the nucleus. C–E, Analyzed images belonging to the time series in A and showing the situation before (C), during (D), and after (E) influx of RanGAP1:YFP into the nucleus. A horizontal measurement line was drawn straight through the images for determination of fluorescence intensity (in F–H) in a five pixel wide ROI (horizontal box with scale). Exemplary square ROIs used for determination of fluorescence intensity over time **Figure 10: (continued)** (in J) are marked in the cytosol (cyt), nucleoplasm (nc), and nucleolus (no). F–H, Profile of the average fluorescence intensity at the selected horizontal ROIs. F belongs to C, G to D, and H to E. I, Overlay of F–H. J, Time-series plot showing changes in distribution of fluorescence over time due to RanGAP1:YFP flux from the cytosol to the nucleoplasm. For five different gonidia, square ROIs were selected as indicated in C–E. Fluorescence intensity measurements were normalized by the half-maximum fluorescence intensity of the nucleoplasm for each data set before the mean intensity of the selected ROIs was determined for each time point. Error bars indicate the standard deviation. The slope of photobleaching within the cellular compartments is displayed with orange lines.

Transformants expressing the *rangap1* gene of *V. carteri* fused to *yfp* (Supplemental Figure S1E) allow for subcellular localization of YFP-tagged RanGAP1 (Figure 10 and Supplemental Movie S9). RanGAP1:YFP resides in the cytosol during interphase, but at late prophase, shortly before the disintegration of the nucleolus, it begins to enter the nucleus of dividing gonidia (Figure 10A). With increasing influx of RanGAP1:YFP into the nucleus, the negatively stained nucleolus becomes visible, at which point it also begins to disintegrate. During metaphase, RanGAP1:YFP is evenly distributed in the nucleoplasm (Figure 10B). Thus, chromosomes and even spindle microtubule bundles are visible as silhouettes. Chromatin decondensation already begins during anaphase, before the chromatids reach the spindle poles (Figure 10B at 03:30 to 04:30). Remodeling of the nuclear envelope structure starts in late anaphase. The nuclear envelope breaks into patchy structures with irregular dark tubules (Figure 10B from 5:30 onward). As already shown in the course of H2B:YFP localization, prolonged chromatid cohesion frequently leads to the formation of a temporary anaphase bridge (Figure 10B from 5:30 onward). The formation of the new nuclear envelopes then starts from the chromatin surface. RanGAP1:YFP is excluded from the daughter nuclei and regains its cytoplasmic localization (Figure 10B at 16:00), which is essential for nuclear trafficking. Quantitative measurements of fluorescence intensity during RanGAP1:YFP influx into the nucleus show that the strongest fluorescence intensity on the cytosolic side is consistently found in the perinuclear cytosol close to the nuclear envelope (Figure 10, F–I). Correspondingly, a continuous, fluorescent perinuclear layer, approximately 0.5 µm thick, is also visible in CLSM time-series images before (Figure 10, C and F), during (Figure 10, D and G), and after (Figure 10, E and H) RanGAP1:YFP influx. We also measured RanGAP1:YFP fluorescence intensity over time (Figure 10J). RanGAP1:YFP reaches its final distribution between the cytosol and nucleoplasm within only about 5 min after the onset of its influx into the nucleus. As expected, the steep increase in fluorescence inside the nucleus of approximately 1.8 rlu is accompanied by only a small decrease in cytosolic fluorescence of approximately 0.2 rlu due to the quite different volumes of cytosol and nucleoplasm. After the end of RanGAP1:YFP relocation, cytosolic fluorescence is approximately 45% of the fluorescence within the nucleus (Figure 10J), which indicates that the RanGAP1:YFP concentrations in the nucleoplasm and cytosol are about the same (Supplemental Figure S5).

### Migration of DRP1 during cell division

**Figure 11:**
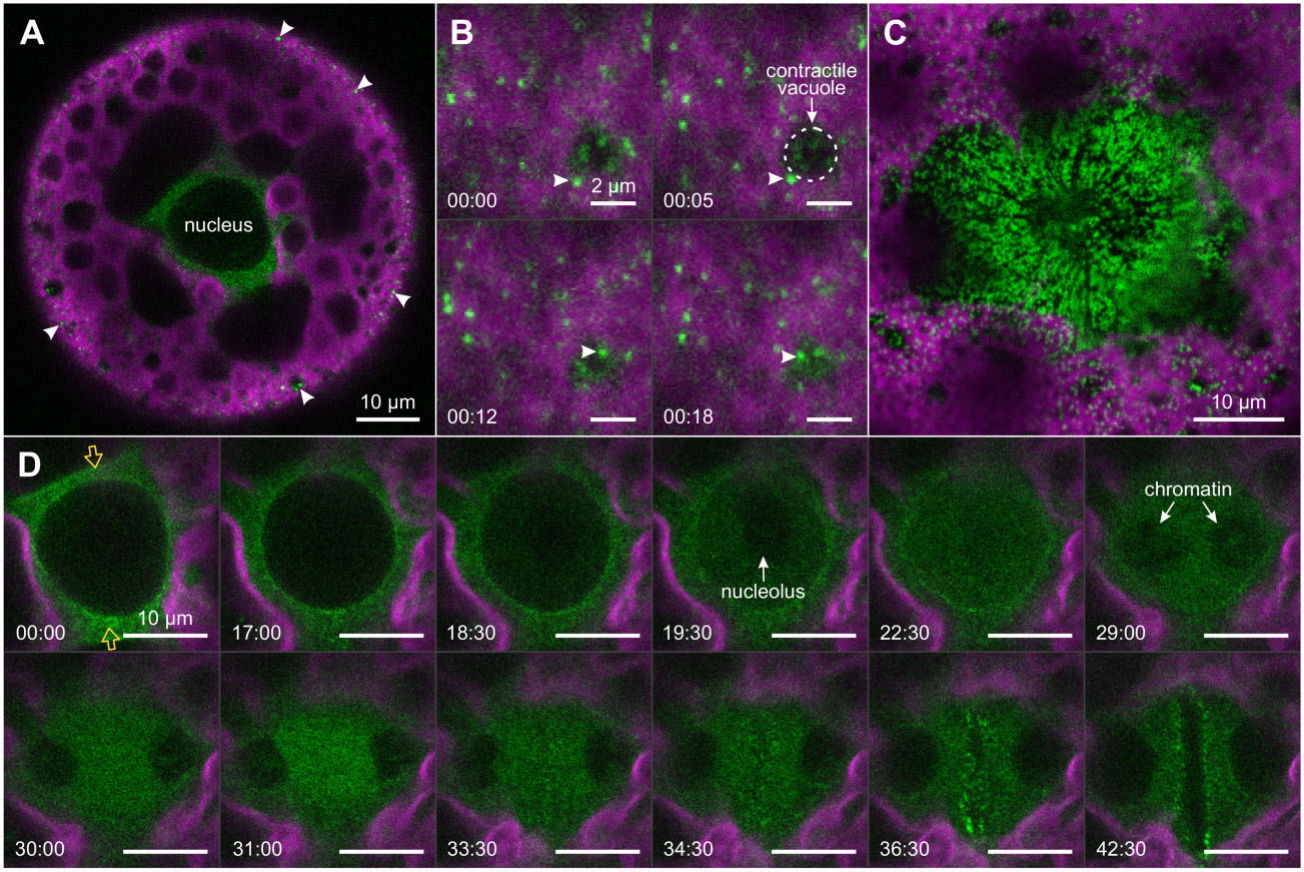
Localization of YFP:DRP1 during the first embryonic mitosis. In vivo CLSM imaging of DRP1 using *Volvox* transformants that produce fluorescent YFP:DRP1 (green). The chlorophyll fluorescence of chloroplasts (magenta) is shown for orientation. A, B, and D, Fluorescence phenotype of representative transformants. C, Fluorescence phenotype of a transformant with strong overproduction of YFP:DRP1. All images show top views onto the nucleus (A, D) or the surface (B, C) of gonidia. A, Optical cross section of a gonidium during preprophase showing YFP:DRP1 fluorescence around the nucleus and at the surface of the cell (white arrowheads). B and D, Time-series images. Time differences in relation to the first image are given in min:s. B, Top view onto the surface of a gonidium. Note that the YFP:DRP1 speckles change their position. One speckle in the vicinity of a contractile vacuole is marked (white arrowhead) as an example. C, Top view onto the anterior surface of a transformant with strong overproduction of YFP:DRP1. Fluorescent speckles accumulate massively above the dividing nucleus. D, Time-series images of a dividing nucleus (same gonidium as in A). The series starts at prophase and ends with the formation of the cleavage furrow in the nuclear plane during cytokinesis. Two orange arrows indicate the position of the division plane.

DRP1 is a membrane-associated protein thought to be involved in membrane remodeling during cytokinesis (Konopka et al., 2006). In transformants expressing the *yfp* coding sequence fused to the *drp1* gene of *V. carteri* (Supplemental Figure S1F), YFP-tagged DRP1 is located in the cytosol (Figure 11). More specifically, YFP:DRP1 is fairly evenly distributed in the cytosol, but it is also found in numerous small speckles located in the cytosol near the cell surface (Figure 11A). The diameter of a single speckle is up to 0.4 µm. In other species, various members of the DRP family are known to be present in the cytosol as dimers, tetramers, or oligomers and to form polymers when recruited to membrane structures (Praefcke and McMahon, 2004; Macdonald et al., 2014; Ramachandran and Schmid, 2018). Therefore, based on their size and their round, regular shape, the bright speckles at the surface of *V. carteri* gonidia could represent membrane vesicles (Bednarek and Backues, 2010) to which YFP:DRP1 is attached. Most of these fluorescent vesicles show irregular movements, which are most evident near the contractile vacuoles because of the strong cytoplasmic streaming there (Figure 11B). In a transformant producing exceptionally high levels of YFP:DRP1, the fluorescent vesicles accumulate above the nucleus during cell division (Figure 11C). The evenly distributed fluorescence in the cytosol (Figure 11, A and D) most likely originates from monomeric YFP:DRP1. At the beginning of mitosis, the YFP:DRP1 that is located around the nucleus accumulates near the division plane (Figure 11D at 00:00 and Supplemental Movie S10), where the microtubule bundles approach the nucleus (Figure 6E). However, at late prophase, it is evenly distributed again (Figure 11D at 17:00). Shortly before the disintegration of the nucleolus, YFP:DRP1 enters the nucleus (Figure 11D at 17:00 to 22:30). In contrast to YFP:NLS and RanGAP1:YFP, YFP:DRP1 is not uniformly distributed between the cytosol and nucleoplasm, which is presumably due to the membrane association of DRP1 causing a slightly higher concentration of YFP:DRP1 in the cytosol (Supplemental Figure S5G). The disintegrating nucleolus and a little later the chromosomes are visible as silhouettes. In telophase, the amount of YFP:DRP1 between the forming nuclei transiently increases (Figure 11D at 31:00). Due to the invagination of the cleavage furrow, the fluorescent YFP:DRP1 speckles near the cell membrane eventually come into the optical section through the nuclear plane (Figure 11D at 34:30 to 42:30).

### A model for mitosis in *V. carteri*

**Figure 12:**
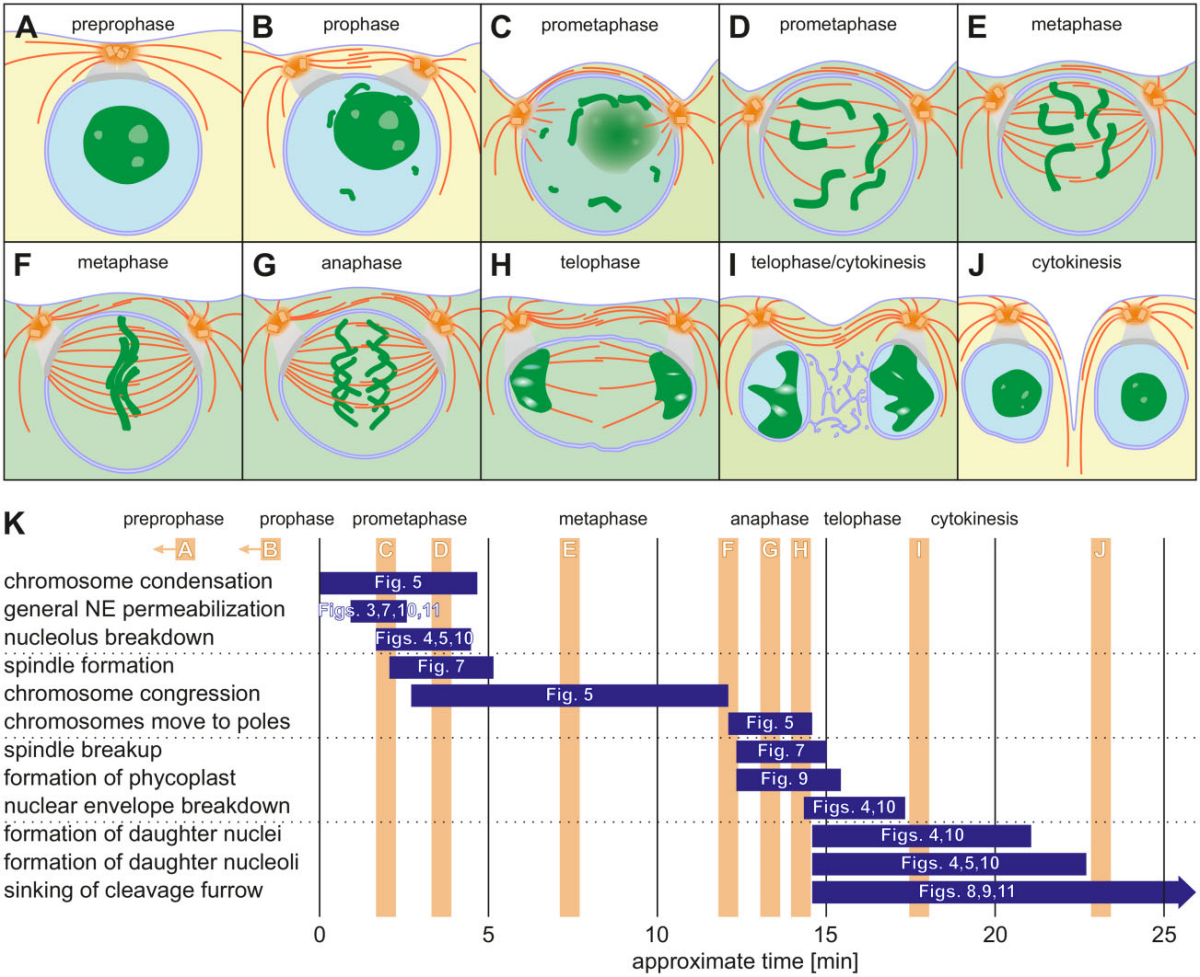
Schematic steps of the first embryonic mitosis in *V. carteri* with temporal assignment and references to fluorescence images. A–J, Schematic representation of the first embryonic cell division as side view cross section showing microtubular structures (orange), chromatin (dark green), NBBCs (gray), and membranes (dark blue). The change in permeability of the nuclear envelope results in temporary mixing of cytosol (yellow) and nucleoplasm (light blue), which is indicated in light green. K, Temporal assignment of the steps of mitosis shown in A–J (vertical orange bars). The length of the purple time bars represents the approximate duration of the processes mentioned on the left side. Figure references on the time bars refer to the corresponding fluorescence images. The beginning of chromosome condensation is at 0 min. A and B occur within a period of 20–30 min before chromosome condensation. NE, nuclear envelope.

Based on our data and previous work, we have developed a model for the first embryonic cell division of *V. carteri* (Figure 12 and Supplemental Figure S11). The first visible sign of commitment to mitosis is the restructuring of the microtubule cytoskeleton in preprophase. The long cytoplasmic interphase microtubules are converted into shorter microtubules and at the same time the number and density of the microtubule bundles increase (Figure 6, A and B). Subsequently, the microtubule aster changes from a radial symmetric arrangement to a bidirectional structure that is aligned to the four-membered microtubular rootlets (Figure 6C). Two microtubule-based structures extend into the cell and enclose the nucleus (Figure 6, D and E). The overall structure of the gonidium also changes significantly. The large vacuoles are restructured into several smaller vesicles. In addition, the nucleus and the basal apparatus/centrosome move much closer together (Figures 2, A–E and 5C). Because the basal apparatus/centrosome is anchored to the cell membrane the nucleus also comes closer to the anterior cell surface (Figure 12A). Based on results in *C. reinhardtii*, this approach of the nucleus to the cell surface is due to contraction of the centrin-rich nucleus-basal body connectors (NBBCs) that connect the basal bodies to the nucleus (Salisbury et al., 1988). This contraction apparently creates a tensile stress between the nucleus and the cell membrane, causing not only the nucleus to move toward the cell membrane, but also the cell membrane to move toward the nucleus. The latter movement is evident from a flattening of the gonidium at the anterior pole (Figure 5C), which eventually results in a rhomboidal pit (Green and Kirk, 1981). The distance between the cell membrane and the nuclear envelope decreases by approximately 20 µm and the nucleus is then only about 5 μm underneath the cell membrane (Figure 5, C and D).

In prophase, the duplicated centrosomes are spatially separated and positioned for spindle formation (Figures 6F and 12B). The process is guided by the microtubular rootlets, as shown in both *Chlamydomonas* and *Volvox* (Holmes and Dutcher, 1989; von der Heyde and Hallmann, 2020). According to results in *C. reinhardtii*, the two-membered rootlets provide orientation, whereas the four-membered rootlets connect the two pairs of centrioles (Doonan and Grief, 1987; Segaar and Gerritsen, 1989; Gaffal and el-Gammal, 1990). In addition, the two separating centrosomes nucleate microtubule asters and are connected by numerous parallel microtubules running along the surface of the nuclear envelope (Figure 6, F–J). The process of centrosome separation is apparently accomplished by these different microtubular structures. The early rotational movements of the centrioles are shaped by interacting microtubular rootlets, whereas the numerous antiparallel and astral microtubules serve to move the centrosomes apart in a straight line. Together, this results in the characteristic shape of the microtubular rootlets (Figure 6H). We would like to emphasize the significance of observing astral microtubules, as the presence of astral microtubules has been questioned in *C. reinhardtii* (Marshall, 2009).

Most of the microtubular connections between the centrosomes are degraded immediately after centrosome separation (Figure 7A), but the centrosomes remain connected via the microtubular rootlets (Figure 12, C–E). The microtubular rootlets as a whole resemble two point-symmetric hooks and constitute the structural basis for the later formation of the phycoplast.

At late prophase, the chromosomes begin to condense (Figures 5 and 12B). Unlike the situation in vascular plants and animals, the nuclear envelope of green microalgae does not break down at this mitotic stage. Nevertheless, the extensive protein exchange between the cytosol and nucleoplasm (Figures 3A, 7B, 10, and 11D) suggests substantial permeabilization of the nuclear envelope leading to loss of nuclear identity during mitosis (Figure 12, B–D). This permeabilization marks the transition from prophase to prometaphase in *V. carteri*. The nucleolus then moves close to the nuclear envelope and disintegrates, while condensing chromosomes become visible (Figures 4, 5, 10, and 12, B–D). The spindle begins to form starting from the centrosomes located immediately outside the nuclear envelope (Figure 7, A–C and 12C). After the initiation of spindle formation, the NBBCs lose their tension and, as a result, the centrosomes move away from the spindle poles (Figure 12, C–F). Microtubules of the forming spindle attach to the kinetochores at the centromeres of the chromosomes.

Once all chromosomes have reached the metaphase plate, anaphase is initiated. Cohesion between sister chromatids is gradually lost (Figures 5 and 12G). The spindle elongates and then its degradation commences (Figures 7C and 12H). Even chromatin decondensation and remodeling of the nuclear envelope are initiated before chromatids reach the spindle poles (Figures 4A, 5A, and 10B). Simultaneously with the disintegration of the spindle, the phycoplast begins to form from the microtubular rootlets. Its formation starts at the centrosomes and then continues along the curved microtubular rootlets toward the division plane (Figures 9A and 12, F–H).

During telophase, a new nuclear envelope forms around each set of decondensed chromosomes by coalescence of membrane vesicles derived from the previous nuclear envelope (Figures 4A and 10B). Furthermore, tubulin molecules released from the spindle appear to be directly recruited to phycoplast formation (Figure 8E and Supplemental Figure S9). Once the phycoplast is formed close to the surface of the cell, the cleavage furrow begins to invaginate at the division plane (Figures 8E, 9, and 12, H–J). The movement of the middle section of the phycoplast toward the posterior end of the gonidium coincides with the progressing invagination of the cleavage furrow and begins even before the spindle is fully degraded (Movie 3). This process generates the typical vertical arrangement of the cleavage microtubules (Figure 9, C and D), which are in parallel to the advancing cleavage furrow and perpendicular to the previous axis of the mitotic spindle. The process probably also positions the microtubular rootlets that mark the next division plane, as can be concluded from studies in *C. reinhardtii* (Holmes and Dutcher, 1989; Ehler and Dutcher, 1998; Kirk, 1998). Remarkably, the first cytokinesis is not completed until after the onset of the second embryonic cell division. Even then, the cells of the embryo remain connected by cytoplasmic bridges (Green and Kirk, 1981).

## Discussion

Three crucial features characterize mitosis in *V. carteri* but are also relevant to other forms of mitosis: (i) the grade of interchange between the cytosol and nucleoplasm, (ii) the restructuring of the nuclear envelope, and (iii) the organization of the spindle formation. These characteristics are mostly linked, as the nuclear envelope mediates nuclear permeability and spindle formation is frequently regulated by the cytoplasmic centrosomes. The nuclear envelope of *V. carteri* surrounds the mitotic nucleus until the daughter nuclei are formed (Figures 4 and 10) and provides a barrier, separating nuclear processes from cytoplasmic processes. However, even after its general permeabilization during mitosis, the barrier function is not completely lost because the nuclear envelope still prevents chromosomes from spreading throughout the cell and cytoplasmic organelles from invading the spindle area. In *V. carteri*, breakdown of the old and assembly of the new nuclear envelopes are combined in a single restructuring event, allowing immediate reuse of the components involved. This also circumvents the disassembly and reassembly of the nuclear pore complexes, which is observed in open mitosis of vascular plants and animals (Kiseleva et al., 2001; Ungricht and Kutay, 2017). Thus, late remodeling of the nuclear envelope is likely to be faster and more energy efficient than the open mitosis. This is supported by the finding that organisms with rapid mitotic divisions usually retain the identity of the nuclear envelope (Makarova and Oliferenko, 2016).

### Loss of nuclear identity during mitosis

During the early mitosis of *V. carteri*, the nuclear envelope becomes leaky (Figures 3, 7, 10, and 11) and equalization of the protein concentrations on both sides of the envelope then takes only about 5 min (Figures 3C and 10J). Cytosol and nucleoplasm are intermixed and nuclear identity is temporarily lost. Such a loss of nuclear identity without nuclear envelope breakdown is otherwise found, for example, in fungi (De Souza et al., 2004; Arai et al., 2010), insects (Kiseleva et al., 2001), and nematodes (Lee et al., 2000). Loss of the nuclear permeability barrier can basically be achieved by three different processes: (i) perforation of the nuclear envelope, (ii) modification of the permeability properties of nuclear pore complexes, and (iii) changes in regulation and specificity of nuclear transport processes. Thus, the question arises in which way the loss of the nuclear permeability barrier is actually achieved in *V. carteri*.

Electron microscopy images indicate that during mitosis membrane-free polar fenestrae are formed in the nuclear envelopes of *V. carteri* and *C. reinhardtii* (Johnson and Porter, 1968; Coss, 1974; Birchem and Kochert, 1979; O’Toole and Dutcher, 2014). In *Caenorhabditis elegans* and *Drosophila melanogaster* such polar fenestrae are required for the interaction of cytosolic centrosomes with the mitotic spindle (Lee et al., 2000; Kiseleva et al., 2001). Because spindle formation in *V. carteri* also originates from cytosolic centrosomes (Figure 7, A–C), although the nuclear envelope persists until late anaphase, it can be assumed that a specific structure reaches through each polar fenestra allowing for interaction and transmission of forces across the nuclear envelope. In the nuclear envelope of two other members of the Chlamydomonadales, namely *Dunaliella* and *Spermatozopsis*, protein structures with a plaque-like shape were identified and spindle microtubules emanate from these structures (Bouck and Brown, 1973; Lechtreck and Grunow, 1999; Grunow and Lechtreck, 2001). Thus, the polar fenestrae are most likely filled and sealed with specific structures that allow interaction and transmission of forces across the nuclear envelope but no substance exchange. Instead, the general permeabilization of the nuclear envelope during mitosis of *V. carteri* is likely achieved by modification of the nuclear pore complexes. In *C. reinhardtii*, electron micrographic studies indicate that nuclear pore complexes are altered during mitosis (Johnson and Porter, 1968). However, the image resolution of these electron micrographs is too low to reveal details and newer studies analyzed only nuclear pores during interphase (Mosalaganti et al., 2018).

Eventually, the barrier function of the nuclear envelope might also be reduced or removed by modifying the transport mechanisms in particular by modulation of the RanGTP gradient across the nuclear envelope. In the fission yeast *Schizosaccharomyces pombe*, this is achieved by nuclear import of a RanGAP1 homolog, which is otherwise always in the cytosol (Melchior et al., 1993), just like in *V. carteri* (Figure 10), *Aspergillus* *nidulans* (De Souza et al., 2004), and other species. Cytosolic RanGAP1 ensures that RanGTP is hydrolyzed only in the cytosol (Macara, 2001), which is essential for both nuclear import and nuclear export. Based on results in fungi, it is believed that translocation of RanGAP1 into the nucleus is a strategy for inducing the collapse of the RanGTP gradient across the nuclear envelope resulting in temporary abolishment of nuclear identity (De Souza and Osmani, 2007; Asakawa et al., 2010; Asakawa et al., 2011). Our finding that *V. carteri* RanGAP1 is also translocated to the nucleus may indicate that this characteristic is conserved among some eukaryotes of the Opisthokonta and Chloroplastida lineages. In particular, this trait appears to be found in unicellular and colonial species and those with a relatively simple morphology and a small number of cell types. Another conserved trait could be that *V. carteri* RanGAP1, unlike its homologs in vascular plants and animals, always remains soluble and has no affinity for the nuclear envelope, spindle, or division plane (Figure 10), which is also evidenced by the absence of the corresponding targeting and binding domains (Supplemental Figure S12). RanGAP1 of the fungi *S. pombe* and *Saccharomyces cerevisiae* is also soluble (Hopper et al., 1990; Melchior et al., 1993) and in both *V. carteri* and fungi, RanGAP1 influx into the nucleus occurs with the membrane structure of the nuclear envelope being largely intact. Because of the similarities in mitosis between these very distantly related species, this type of mitosis could be classified as an ancient form of mitosis, which then evolved independently into the open forms of mitosis in vascular plants and animals.

### The role of centrosomes in spindle formation

Throughout the Eukaryota, the formation of the spindle is induced and regulated by various pathways. Remarkably, spindle formation in vascular plant and acentrosomal animal cells does not require centrosomes (Heald et al., 1996; Clift and Schuh, 2015; Joukov and De Nicolo, 2019; Hoffmann, 2021). In animal cells, chromosome-driven spindle assembly based on local RanGTP gradients provides an alternative or a complement to the involvement of centrosomes (Wilde and Zheng, 1999; Caudron et al., 2005; Wu et al., 2013; Kapoor, 2017). However, in *V. carteri*, there is apparently a significant involvement of centrosomes. Before mitosis, the nucleus and the basal apparatus/centrosome, which is anchored to the cell membrane, move quite close together (Figures 2, A–E and 5C) and, thus, the nucleus comes close to the surface of the cell. The benefit of this energy-consuming relocation of a large organelle is apparently greater than the disadvantage due to proximity to the cell surface, which reduces the protection provided by the pigments of the chloroplasts and thus exposes the DNA to more damaging UV light during mitosis. Before spindle formation (Figure 7, A–C), the centrosomes separate from each other (Figure 6, F–J) to then primarily determine the origin of the spindle poles. In animals like *Drosophila* and *Caenorhabditis*, centrosomes are essential for the rapid progression of embryonic cell divisions (Meraldi, 2016; Hoffmann, 2021), which may suggest that centrosomes also provide a speed advantage in palintomy of green microalgae. The correct positioning of animal centrosomes prior to the nuclear envelope breakdown increases the efficiency and reliability of chromosome attachment, while defects concerning the centrosome separation can in turn lead to aneuploidy and ultimately cell death (Silkworth et al., 2012; Agircan et al., 2014; Meraldi, 2016; Stiff et al., 2020; Hoffmann, 2021).

Regarding green microalgae, it was previously even unclear whether their centrosomes form microtubule asters, because these microtubule structures could not be detected in fixed specimen (Ehler et al., 1995; Marshall, 2009). In vivo visualization of microtubules by fluorescence tagging of a microtubule-binding protein (EB1) could not reveal the fate of the centrosomes during mitosis, as EB1 seemed to disappear from the centrosomes as cells entered mitosis and solely stained the mitotic spindle (Onishi et al., 2020). However, our data show that for centrosome separation in *Volvox*, in addition to microtubular rootlets, distinct microtubule asters and an array of antiparallel microtubules form between the separating centrosomes (Figure 6G). These microtubular structures strongly resemble the cytoskeleton involved in centrosome separation in animal cells (Agircan et al., 2014; Stiff et al., 2020). Therefore, they are most likely involved in moving the centrosomes apart, as in animals. In animals, the microtubule array that drives centrosome separation is commonly understood to be part of spindle formation, as the two processes are closely intertwined, greatly facilitated by nuclear envelope breakdown (Tanenbaum and Medema, 2010; Hoffmann, 2021). In contrast, there is no nuclear envelope breakdown in *Volvox*. Therefore, the *Volvox* spindle represents another, intranuclear microtubule array, which is spatially separate from the cytoplasmic microtubule array and which also arises later in time (Figure 7, A–C). Spindle formation in *Volvox* originates very close to the centrosomes (Figures 7B and 12C), although the centrosomes are on the outer surface of the nuclear envelope and the spindle formation is initiated near to the inner surface of the nuclear envelope. However, since this is exactly where the polar fenestrae are located in the nuclear envelope (O’Toole and Dutcher, 2014), interaction and force transmission between the two sides is possible through them. By defining the origin of the spindle poles, the orientation of the spindle is determined. After initiation of spindle formation, the centrosomes move away from the nuclear envelope and thus also from the spindle poles. Between spindle and microtubule asters even an area without tubulin develops and enlarges (Figure 7). The centrosomes are obviously not needed for the complete development and shaping of the spindle. An accurate alignment of the spindle is important for cell division of any (multicellular) eukaryote, with large cells, as in *Volvox*, presenting a particular challenge. *Volvox* manages the alignment of the spindle and division furrow with the participation of centrosomes.

### An evolutionary view on mitosis in volvocine algae

Centrosome separation in green microalgae such as *V. carteri* involves both pronounced microtubule asters and an array of antiparallel microtubules between the centrosomes. Thus, this process is much more similar to the centrosome separation in animal cells than previously thought. In vascular plants, these microtubular structures do not exist. This trait therefore appears to be conserved in green algae and animals, whereas it is no longer present in vascular plants in this form. Instead, vascular plants evolved plant-specific microtubular structures (Buschmann and Zachgo, 2016).

In green microalgae, the correct positioning of centrosomes is essential for spindle formation. The coordination of intranuclear spindle formation is carried out by the cytoplasmic centrosomes, although the nuclear envelope lies in between. In contrast, spindle formation in vascular plant and acentrosomal animal cells does not require centrosomes (Heald et al., 1996; Clift and Schuh, 2015; Joukov and De Nicolo, 2019; Hoffmann, 2021). In centrosomal animal cells, the centrosomes are also involved in spindle alignment, as are the spindle pole bodies, the functional equivalents of centrosomes, in yeast (Conduit et al., 2015; Pineda-Santaella and Fernandez-Alvarez, 2019; Hoffmann, 2021). Overall, this suggests that centrosomes were already involved in spindle guidance in the last common ancestor of Chlamydomonadales and Opisthokonta. In centrosomal cells of recent animals, centrosomes are not essential for spindle formation (Brunet et al., 1998; Ohba et al., 1999; Wilde and Zheng, 1999; Megraw et al., 2001; Fant et al., 2004). Centrosomes therefore appear to have lost importance for spindle formation during the evolution of animals and vascular plants, whereas they are still essential for it in green microalgae. Nevertheless, neither in the early phase of the evolution of eukaryotes nor today, centrosomes are not passive “passengers” in cell division (Debec et al., 2010) but take an active role in mitosis.

Although the nuclear envelope does not break down during early mitosis of green microalgae, there is an extensive interchange between the cytosol and nucleoplasm resulting in loss of nuclear identity. In contrast, in early mitosis of animals and vascular plants, there is a complete breakdown of the nuclear envelope and complete intermixing (Kiseleva et al., 2001; Ungricht and Kutay, 2017). As a consequence, open mitosis also requires the degradation and reassembly of the nuclear pore complexes (Kiseleva et al., 2001; Ungricht and Kutay, 2017). Late remodeling of the nuclear envelope in green microalgae appears to require only translocation of nuclear envelope components, which seems to be advantageous for rapid mitotic divisions. Relative to open mitosis in animals and vascular plants, the closed form with loss of nuclear identity, found in green microalgae, appears to be more primordial (see above).

In cytokinesis, the phycoplast is a microtubule structure typical for green algae, whereas in vascular plants there is the phragmoplast. A major difference between the two is that in the phycoplast the orientation of microtubules is parallel to the plane of cleavage and in the phragmoplast it is perpendicular to the plane of cleavage (Marshall, 2009). Although, evolutionarily, the phragmoplast first appeared in advanced green algae (Pickett-Heaps, 1975; Graham et al., 2000), which at least nowadays usually have a phycoplast, the underlying evolutionary patterns remain nebulous and require further investigation.

Especially during interphase, the conserved trafficking protein RanGAP1 helps transport other proteins between the cytoplasm and nucleus. In vascular plants and animals, the protein is bound to the nuclear envelope via specific binding domains. Plant RanGAP1 is targeted to the nuclear rim (Rose and Meier, 2001) and in animal cells, RanGAP1 is anchored to the outer basket of the nuclear pore (Ohtsubo et al., 1989). In contrast, RanGAP1 from green microalgae such as *V. carteri* has no targeting and binding domains (Supplemental Figure S12) and always remains soluble (Figure 10). The soluble RanGAP1 could be an ancient form, also because it was found to be soluble in unicellular fungi (Hopper et al., 1990; Melchior et al., 1993).

In summary, *V. carteri* is a particularly well-suited system for studying mitosis in green microalgae. When compared with mitosis in distantly related organisms, it becomes clear that characteristic components of the quite different animal, plant, and fungal forms of mitosis can be identified in the embryonic cell divisions of *V. carteri*. However, the evolutionary history of the various existing solutions for nuclear division is still relatively vague because previous studies mostly used yeasts, amoeboid organisms, or animal and plant cells (De Souza and Osmani, 2007; Arnone et al., 2013; Boettcher and Barral, 2013; Sazer et al., 2014), thus only partially covering the domain of Eukaryota. Further analyses of *V. carteri* mitosis can usefully complement such evolutionary research by representing simple multicellular eukaryotes from the chlorophyte lineage.

## Materials and methods

### Strains and culture conditions

The wild-type *V.* *carteri f. nagariensis* strain Eve10 (female) (Starr, 1969, 1970; Adams et al., 1990; Kianianmomeni et al., 2008), which originates from Japan, was used to generate the non-revertible nitrate-reductase deficient (*nit*A^−^) strain TNit-1013 (Huskey et al., 1979; Tian et al., 2018). A deletion of 1013 bp in the nitrate reductase (*nitA*) gene prevents strain TNit-1013 from growth in medium that contains nitrate as the sole source of nitrogen. Strain TNit-1013 was used as a recipient strain for transformation experiments and was grown under asexual conditions in standard *Volvox* medium (Provasoli and Pintner, 1959; Starr, 1969) supplemented with 1 mM ammonium chloride as a nitrogen source. For selection of transformants, the defective *nit*A gene of TNit-1013 was complemented with an intact *nitA* gene using plasmid pVcNR15 (Gruber et al., 1996) and growth was in standard *Volvox* medium without ammonium chloride. Synchronous algae cultures were grown at 28°C in a cycle of 8-h dark/16-h cool fluorescent white light (Starr and Jaenicke, 1974) at an average of ∼100 µmol photons m^−2^ s^−1^ photosynthetically active radiation in glass tubes or Fernbach flasks. The glass tubes had caps that allow for gas exchange and the Fernbach flasks were aerated with approximately 50 cm^3^ sterile air/min. The culture density was always kept below 10 spheroids per milliliter to allow for optimal growth and synchronicity.

### Identification of suitable genes

Protein candidates were found through literature search and the corresponding *Volvox* genes were then identified by TBLASTN searches (Altschul et al., 1990) against the genome of *V. carteri* (Prochnik et al., 2010) in Phytozome 12 (Goodstein et al., 2012). The predicted coding sequences were aligned with available RNAseq data (Klein et al., 2017) and both gene models and sequences were corrected where necessary. If there were multiple gene copies in the *V. carteri* genome, literature information and RNA-Seq expression data were considered to identify the most promising gene copy. Literature information was also used to decide whether N- or C-terminal fusions are more promising. All expression vectors constructed for this study contain the nucleotide sequence encoding the yellow fluorescent protein mVenus (YFP) (GenBank acc. no. AAZ65844) (Kremers et al., 2006). The *yfp* gene version used was previously engineered to match the codon usage of *C. reinhardtii* (Lauersen et al., 2015) but it is also suitable for use in *V. carteri* (Tian et al., 2018; von der Heyde and Hallmann, 2020). For visualization of cell nuclei, YFP was tagged with a minimal nuclear targeting sequence using the NLS of simian virus 40 (PKKKRKV) (Kalderon et al., 1984). Fluorescence tagging of histone H2B was used to stain chromatin, as implemented before (Kanda et al., 1998; Hirakawa et al., 2011). Gene Vocar.0027s0143 (Genbank: M31922.1) was selected from 13 annotated *h2b* genes found in the *V. carteri* genome (Müller et al., 1990; Prochnik et al., 2010). YFP was fused to the N-terminus of *V. carteri* β-tubulin TubB2 (Vocar.0007s0229) (Harper and Mages, 1988; Prochnik et al., 2010) to enable the visualization of microtubules. In *V. carteri*, RanGAP1 is encoded by a single-copy nuclear gene (Vocar.0048s0050) (Prochnik et al., 2010). In the course of the verification of this gene, we also compared and aligned the *V. carteri* RanGAP1 protein sequences with RanGAP1 protein sequences of completely different origin (Supplemental Figure S12). For the *rangap1* gene, we constructed two expression vectors encoding N- and C-terminally tagged RanGAP1 because the literature information regarding the best fusion site was insufficient and we wanted to reduce the likelihood that YFP tagging affects the natural protein localization of RanGAP1. Since later N- and C-terminally labeled RanGAP1 showed the same localization, we continued our experiments only with one variant, the C-terminally tagged RanGAP1. The gene of DRP1 (Phytozome ID Vocar.0026s0065) (Prochnik et al., 2010) was verified by comparison with related dynamin genes and by alignments of the *V. carteri* DRP1 protein sequence with DRP1 protein sequences of related species.

### Sequence comparisons

Protein sequences were aligned where appropriate using the MUltiple Sequence Comparison by Log-Expectation program (MUSCLE) (Edgar, 2004). Management of multialigned data was done using BioEdit 7.2 (Hall, 1999). Alignments were illustrated using GeneDoc 2.7 (Nicholas et al., 1997). Phylogenetic relations of selected species in Supplemental Figure S12A were taken from previous publications (Kuramae et al., 2006; Hallmann, 2011; Leonard and Richards, 2012).

### Construction of vectors for expression of fusion proteins in *V. carteri*

For construction of expression vectors (Supplemental Figure S1 and Supplemental Data Set S3), DNA fragments were amplified by recombinant PCR using the oligonucleotides listed in Supplemental Table S2. Oligonucleotide primers were designed using the primer analysis software OligoCalc (Kibbe, 2007) and Primer-BLAST (Ye et al., 2012). Genomic DNA was extracted from *V. carteri* using the DNeasy Plant Mini Kit (Qiagen, Hilden, Germany). For extraction of total RNA, frozen *V. carteri* algae were homogenized in a Precellys Evolution bead mill homogenizer (Bertin Technologies, Montigny Le Bretonneux, France). RNA was extracted from these lysates using phenol-based TRI Reagent (Sigma-Aldrich, St. Louis, MO, USA) and trichloromethane. RNA was precipitated and purified as previously described (Lerche and Hallmann, 2009). The purity and quantity of DNA and RNA were checked using agarose gel electrophoresis and a NanoPhotometer N60 (Implen, Munich, Germany). Reverse transcription was carried out using the RevertAid First Strand cDNA Synthesis Kit (Thermo Fisher Scientific, Waltham, MA, USA). PCR reactions with genomic DNA or cDNA as a template were performed as specified by the manufacturer using a Phusion High-Fidelity DNA Polymerase (Thermo Fisher Scientific) and a gradient PCR thermal cycler (Applied Biosystems ProFlex PCR System, Thermo Fisher Scientific). The PCR products were cloned and sequenced. For the very large genes of *rangap1* and *drp1*, genomic DNA was combined with cDNA fragments to avoid unnecessary introns (Supplemental Figure S1). Genomic and cDNA fragments were fused using an endogenous *Acc*I restriction site for *rangap1* and an endogenous *Eco*32I restriction site for *drp*1. The final *rangap1* and *drp1* constructs still contain the first two and four introns, respectively, because intronless constructs can show low expression (Gruber et al., 1996). For most of the chimeric genes, the promoter of the *lhcbm*1 gene (Phytozome ID Vocar.0001s0479) was used, which was previously used in *V. carteri* transformants (Klein et al., 2017; Tian et al., 2018). Since tubulin expression driven by the *lhcbm*1 promoter previously did not yield any viable *V. carteri* transformants (von der Heyde and Hallmann, 2020), the endogenous tubulin promoter was used for this expression vector. The terminator region was derived from the *V. carteri lhcbm*1 gene (Vocar.0001s0479) for all constructs. A short linker sequence, which codes for a flexible pentaglycine interpeptide bridge, was inserted between the gene of interest and the *yfp* cDNA (Chen et al., 2013). All constructs were assembled in pBluescript II SK (−) vectors (Agilent Technologies, Santa Clara, CA, USA) using *Escherichia* *coli* strain DH5α as a host. Plasmid vectors were extracted from *E. coli* using the GeneJET Plasmid Miniprep Kit (Thermo Fisher Scientific) and confirmed by sequencing.

### Stable nuclear transformation of *V. carteri*

The *nit*A-deficient *V. carteri* strain TNit-1013 was grown in larger scale in standard *Volvox* medium supplemented with 1 mM ammonium chloride as a nitrogen source. Three milligrams of gold microprojectiles (1.0 µm in diameter, Bio-Rad, Hercules, CA, USA) were coated both with 5 µg of the *nit*A-containing vector pVcNR15 (Gruber et al., 1996) and 5 µg of one of the vectors that encode a fluorescent protein for subcellular localization (Supplemental Figure S1). Vector pVcNR15 contains a modified but intact *V. carteri nitA* gene (Gruber et al., 1996) and, thus, allows for selection of transformants because it is able to complement the *nitA* mutation of strain TNit-1013. DNA coating of microprojectiles was as previously described (Lerche and Hallmann, 2009, 2013). About 24,000 algae were harvested on a 40-µm stainless steel mesh and washed thoroughly with 3 L of standard *Volvox* medium lacking ammonium chloride. Transformation was performed using a Biolistic PDS-1000/He (Bio-Rad) particle gun. The transformation procedure was as previously described (Hallmann and Wodniok, 2006; Lerche and Hallmann, 2009, 2013, 2014) with minor modifications. After the transformation procedure, the algae were incubated under standard conditions including standard *Volvox* medium lacking ammonium chloride, which only allows growth of transformants. From the fifth day on, algae cultures were examined for green and living transformants (*nit*A^+^) in a background of numerous bleaching, unaltered organisms (*nit*A^−^). Identified transformants were transferred to glass tubes containing 10 mL fresh medium for further cultivation. Expression of the co-transformed *yfp* gene constructs was verified by fluorescence microscopy. Transformants showing distinct fluorescence but otherwise having an unaltered wild-type phenotype were used for further analyses (Supplemental Figure S2). For each of the six analyzed proteins, we generated at least three independent transformed strains.

### Preparation of total RNA

*Volvox* spheroids were collected on a 100-μm mesh nylon screen 4 h before onset of the dark phase of the 8-h dark/16-h light cycle, which corresponds to the stage just before onset of embryogenesis. The concentrated algae were frozen in liquid nitrogen and stored at −70°C. Approximately 250 μL of concentrated, frozen algae were disrupted in 2-mL tubes containing 10 1-mm silica beads (Fisher Scientific) in 1 mL of phenol-based TRI Reagent (Sigma-Aldrich) using a tissue homogenizer (Precellys Evolution Homogenizer; Bertin Instruments). Cells were disrupted by running three cycles of 20 s at 10,000 rpm with a pause of 10 s between cycles. For extraction of total RNA, 300 μL of trichloromethane was added to the homogenate. RNA precipitation and RNA purification were as previously described (Lerche and Hallmann, 2009, 2013, 2014). The purity of RNA was determined by measuring the 260/280 and 260/230 ratios using a NanoPhotometer N60 (Implen) UV–Vis spectrophotometer. The RNA was quantified by absorbance at 260 nm. The integrity of RNA was verified by agarose gel electrophoresis. To remove possible contaminating genomic DNA from the purified RNA samples, the samples were treated with DNase I according to the manufacturer’s instructions (Thermo Fisher Scientific).

### RT-qPCR

The SensiFAST SYBR Hi-Rox One-Step Kit (Bioline) and a CFX96 Touch Real-Time PCR Detection System (Bio-Rad) were used for RNA quantification by RT-qPCR. All RT-qPCR experiments were carried out using three biological replicates, that is independent algae cultures. In addition, each biological replicate was analyzed in three technical replicates. The specific primers for amplification of a fragment of *h2b* (Vocar.0027s0143) were 5′-ATACCGGCATCAGCTCGAAG and 5′- CGCACAGCAGTCTGAATTTCG, for *tubb2* (Vocar.0007s0229) they were 5′-CCACATTCAGGGTGGCCAG and 5′-ACATAGCGGCCGCCAGTTG, for *rangap1* (Vocar.0048s0050) 5′-AGAGCTTAGCTCTGAGGTTGC and 5′-CACCTAGCTTCCTAGCGTACG, and for *drp1* (Vocar.0026s0065) they were 5′-TCCCAGCTTTGAGGACATTCG and 5′-CTTTGTAAGGCCTGGCATGTC. For reasons of comparability, the primers used match exactly both the mRNA of the naturally present gene as well as the mRNA of the additionally integrated gene fusions with *yfp*. The gene of the eukaryotic translation elongation factor 1α2 (*eef1*) was utilized as a reference using the primers 5′-GACGATTGCATGCACCACTAAG and 5′-ATCAGCAGGCACACATCAGC (Kianianmomeni and Hallmann, 2013). Reverse transcription was carried out at 45°C for 15 min. Amplification was performed in 40 cycles of 95°C for 7 s, 58°C for 12 s, and 72°C for 7 s. Melting curves were recorded and PCR products were analyzed by gel electrophoresis to verify amplification of a single specific product of the correct size. The relative expression level was calculated using the 2^−ΔCt^ method (Bustin, 2000; Pfaffl, 2001).

### Confocal laser scanning microscopy

For life cell imaging, algae were synchronously grown under standard conditions, harvested on a 100-µm mesh, and transferred with a small amount of standard *Volvox* medium to an uncoated coverslip-bottom culture dish (Ibidi, Germany). A coverslip with four small dots of desiccator grease at the corners, which serve as a spacer, was cautiously placed on the specimen to slightly fix the algae, while preventing excessive compression. Specimens were freshly prepared and examined immediately using an inverted LSM780 confocal laser scanning microscope (Carl Zeiss GmbH, Germany) equipped with a LCI Plan Neofluar 63×/NA 1.3 objective (Carl Zeiss GmbH). A preheated incubation tool was used to keep the algae in the culture dish constantly at the standard temperature of 28°C. The exposure to laser light was kept as low as possible. Fluorescence of both YFP and chlorophyll was excited by an argon-ion (Ar^+^) laser at 514 nm. The emitted YFP fluorescence was detected at 517–553 nm, whereas chlorophyll fluorescence was detected at 651–700 nm. Fluorescence intensities were recorded for YFP and chlorophyll in two channels simultaneously. At the same time, transmission images were generated in a third channel using a transmission-photomultiplier tube (PMT) detector. Images were recorded with 12-bit depth using the ZEN black imaging software (ZEN 2011, Carl Zeiss GmbH, Germany).

### Image processing and analysis

Image processing and analysis were performed using Fiji (ImageJ 1.51w) (Schindelin et al., 2012) and the Fiji plugins MultiStackReg (RID:SCR_016098), StackReg, and TurboReg (Thevenaz et al., 1998). For the analysis of CLSM-time series shown in Figures 3C and 10J and Supplemental Figures S6 and S9, the corresponding time scales were aligned according to the maximum slopes of the curves showing the changes of fluorescence over time for the representative regions of interest (ROIs). The fluorescence intensity of images was normalized by the half-maximum fluorescence intensity of the brightest subcellular compartment of the image series. Mean and standard deviation of the fluorescence intensities were determined for each point in time. The decrease of fluorescence intensity due to photobleaching was approximated by exponential regression lines using the first 5–9 points of a time series. Bleach correction was applied to the time-series images in Figures 4, 5A, 7C, 8E, 10B, and 11D. Figure 7C and Movie 3 were gamma adjusted with a value of 0.7 to improve the overall visibility of microtubular structures. Concerning longer time series, the corresponding figures display representative images. Movies 1 and 2 and Supplemental Movies S1–S6, S9, and S10 were created from all images of the time-series shown in Figures 3A, 4A, 5A, 7, C, D–F, 8E, 9A, 10A, 10B, and 11D and Supplemental Figure S6. The fluorescence profiles in Figure 10, F–I were generated as column average plots by averaging the fluorescence intensity of the five pixels of the vertical width of the ROI and plotting it against the horizontal position of the pixel.

For those bar or line plots where the number of data points for at least one sample or calculation was <6 (Figures 3C and 10J and Supplemental Figures S4, S5G, S6C, and S9B), all data points (raw data and calculations) are listed individually in Supplemental Data Set S4.

## Accession numbers

Sequence data for genes and proteins mentioned in this article can be found at Phytozome or GenBank/EMBL databases under the following accession numbers: YFP (AAZ65844), H2B (Vocar.0027s0143, M31922), TubB2 (Vocar.0007s0229), RanGAP1 (Vocar.0048s0050), DRP1 (Vocar.0026s0065), and LHCBM1 (Vocar.0001s0479).

## Supplemental data

The following materials are available in the online version of this article.

**Supplemental Figure S1.** Expression vectors used for in vivo fluorescence tagging in *V. carteri*.

**Supplemental Figure S2.** Phenotypes of wild-type, recipient, and transformant *V. carteri* strains.

**Supplemental Figure S3.** Analysis of growth behavior of wild-type, recipient, and transformant *V. carteri* strains.

**Supplemental Figure S4.** mRNA expression analysis of target genes in wild-type and transformant *V. carteri* strains.

**Supplemental Figure S5.** Distribution of YFP fusion proteins and pts-free YFP between cytosol and nucleoplasm.

**Supplemental Figure S6.** Dynamics of nuclear efflux and influx of YFP:NLS.

**Supplemental Figure S7.** Repeated nuclear efflux and influx of YFP:NLS.

**Supplemental Figure S8.** Schematic representation of microtubule asters and spindles during the second and third embryonic cell divisions.

**Supplemental Figure S9.** Dynamics of the microtubule cytoskeleton during spindle degradation and phycoplast construction.

**Supplemental Figure S10.** Time-series images of microtubular structures and membrane invagination of the cleavage furrow during the second cell division.

**Supplemental Figure S11.** Assignment of the various figures to the individual steps of the first embryonic mitosis in *V. carteri*.

**Supplemental Figure S12.** Phylogenetic tree of several model organisms with RanGAP1 proteins, structure of their RanGAP1 domains, and sequence alignment of the corresponding RanGAP1 proteins.

**Supplemental Table S1.** Overview of replications in terms of coverage of the mitotic phases sorted by the fluorescent protein used.

**Supplemental Table S2.** Primers for construction of expression vectors.

**Supplemental Data Set S1.** Overview of replications when observing a specific structure, process, or topology using a specific fluorescent protein.

**Supplemental Data Set S2.** Measurements of length, distance, diameter, speed, and angle.

**Supplemental Data Set S3.** Details of constructed expression vectors used for in vivo fluorescence tagging in *V. carteri*.

**Supplemental Data Set S4.** Raw data and calculations for bar or line charts where the number of data points for at least one sample or calculation was <6.

**Supplemental Data Set S5.** Multiple sequence alignment of the RanGAP1 proteins used in Supplemental Figure S12.

**Supplemental Movie S1.** Efflux dynamics of nuclear YFP:NLS at prophase of the first embryonic cell division.

**Supplemental Movie S2.** Efflux and influx dynamics of nuclear YFP:NLS during the first embryonic cell division.

**Supplemental Movie S3.** Formation of the spindle apparatus and structure of the microtubule cytoskeleton during cytokinesis visualized by YFP:TubB2.

**Supplemental Movie S4.** 3D topology of microtubule asters and spindle apparatus after their detachment during metaphase.

**Supplemental Movie S5.** Microtubule-based structures of the phycoplast during the first embryonic cell division.

**Supplemental Movie S6.** Topology of microtubule asters, spindles, and phycoplasts during the second embryonic cell division.

**Supplemental Movie S7.** 3D topology of the phycoplast during early cytokinesis of the first embryonic cell division.

**Supplemental Movie S8.** 3D topology of the phycoplast during advanced cytokinesis of the first embryonic cell division.

**Supplemental Movie S9.** Localization of RanGAP1:YFP during the first embryonic mitosis.

**Supplemental Movie S10.** Localization of YFP:DRP1 during the first embryonic mitosis.

## Supplementary Material

koac004_Supplementary_Data

## Notes

E.L.v.d.H. and A.H. conceived and designed the approach. E.L.v.d.H. performed and conducted the experiments and analyses in coordination with A.H.. Both authors wrote the manuscript and approved the final version of the manuscript.

The author responsible for distribution of materials integral to the findings presented in this article in accordance with the policy described in the Instructions for Authors (https://academic.oup.com/plcell) is: Armin Hallmann (armin.hallmann@gmx.de).
