# Supplementary material for: Molecular and cellular dynamics of early embryonic cell divisions in Volvox carteri
Source: Plant Cell. 2022 Jan 9;34(4):1326–53. doi: 10.1093/plcell/koac004 (PMC9026201; doi:10.1093/plcell/koac004)
Supplement: koac004_Supplementary_Data [file koac004_Supplementary_Data.zip › Supplemental Figures and Tables.pdf]

Supplemental Figure S1.

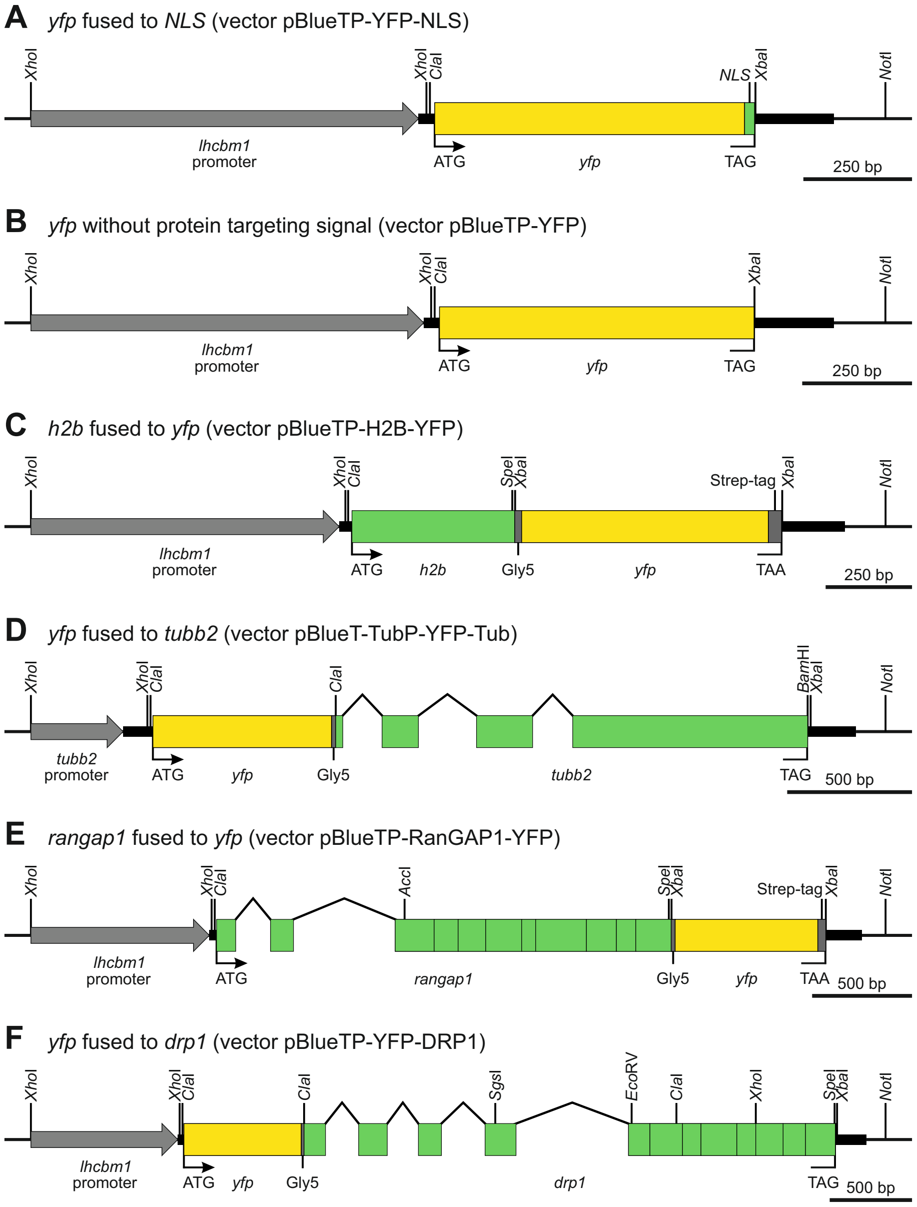

Expression vectors used for *in-vivo* fluorescence tagging in *V. carteri*. Supports Figures 2 to 11. (**A**) Vector for expression of the *yfp* coding sequence fused to a sequence coding for a nuclear localization signal (NLS) of SV40. (**B**) Vector for expression of the *yfp* coding sequence without any protein targeting signal (pts-free YFP). (**C**) Vector for expression of the *V. carteri h2b* coding sequence fused to the *yfp* coding sequence. (**D**) Vector for expression of the *yfp* coding sequence fused to the *V. carteri tubb2* gene. The chimeric gene is driven by the endogenous *V. carteri tubb2* promoter. (**E**) Vector for expression of the *V. carteri rangap1* gene fused to the *yfp* coding sequence. The *rangap1* sequence contains only the first two introns of *rangap1*, whereas the other eight introns were avoided by using cDNA to keep the size of the vector small. (**F**) Vector for expression of the *yfp* coding sequence fused to the *V. carteri drp1* gene. The

drp1 sequence contains only the first four introns of *drp1*, whereas the other seven introns were avoided by using cDNA. (**C** to **F**) Short linker sequences coding for a flexible pentaglycine interpeptide bridge (Gly5) were inserted between the corresponding genes of interest and *yfp*. (**A** to **F**) Promoter regions are represented by grey arrows. In all expression vectors except for the *yfp*:*tubb2*-containing vector, the endogenous *V. carteri lhcbm1* promoter was used. The terminator regions were also derived from the *V. carteri lhcbm1* gene. The 5’ and 3’ untranslated regions are represented by bold black lines. Angled lines depict intron sequences within genomic DNA and vertical lines indicate the position of introns within sequences that were derived from cDNA. The positions of start (ATG) and stop (TAG, TAA) codons are indicated. Restriction sites of enzymes that were used for cloning are specified. All vector backbones were derived from pBluescriptII SK (−), but are not shown in this diagram.

Supplemental Figure S2.

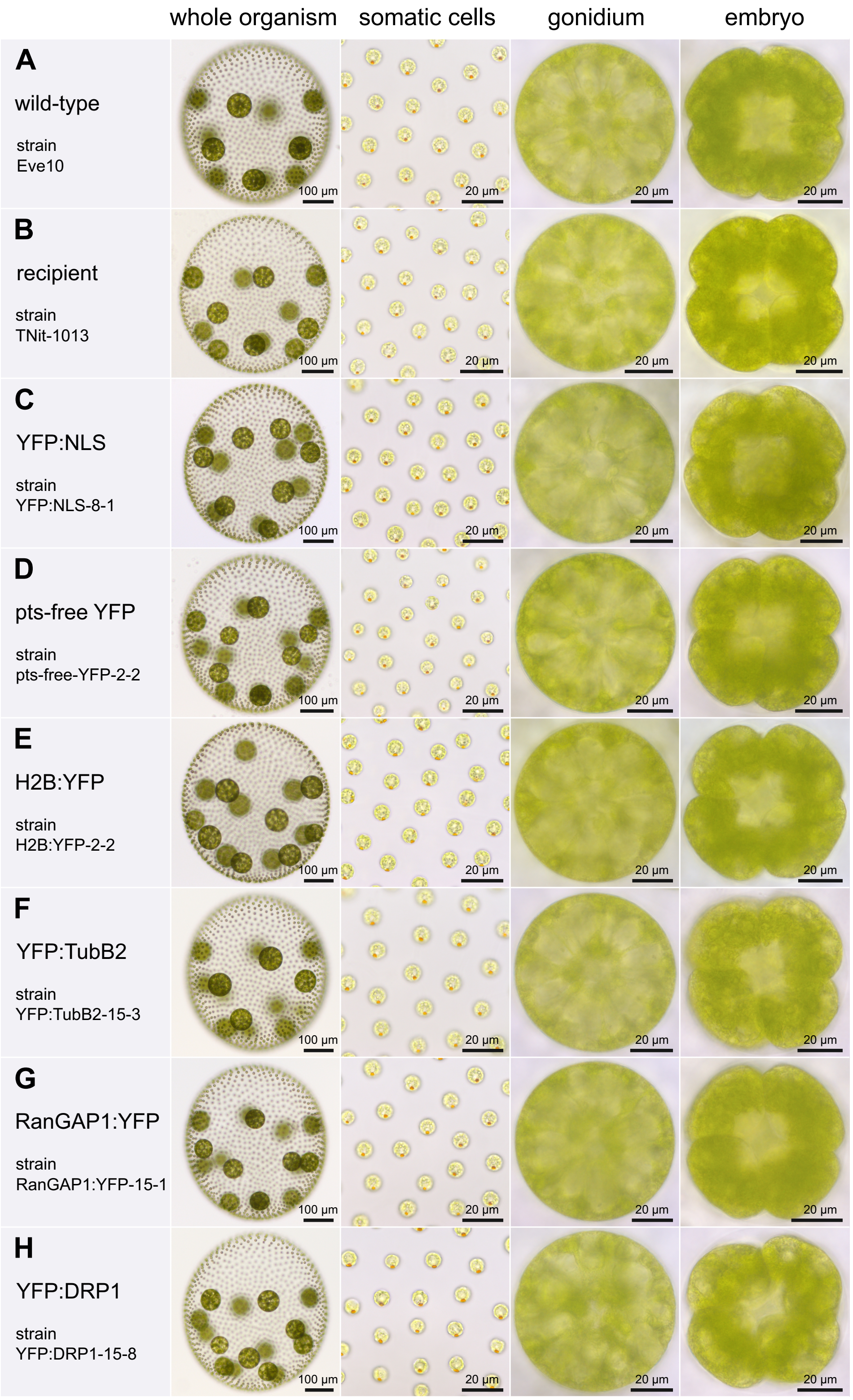

Phenotypes of wild-type, recipient and transformant *V. carteri* strains. Supports Figures 2 to 11. For each strain, the phenotype of the whole organism and enlarged views of some somatic cells, a gonidium and an embryo are shown. Micrographs of whole organisms, somatic cells and gonidia were taken 4 h before onset of the dark phase of the 8 h dark/16 h light cycle, which corresponds to the stage just before onset of embryogenesis. Micrographs of embryos were taken 1.5 hours before onset of the dark phase, which corresponds to the 4-cell stage. (**A**) Wild-type (strain Eve10). (**B**) Nitrate-reductase deficient recipient (strain TNit-1013). (**C**) Transformant producing YFP:NLS (strain YFP:NLS-8-1). (**D**) Transformant producing pts-free YFP (strain pts-free-YFP-2-2). (**E**) Transformant producing H2B:YFP (strain H2B:YFP-2-2). (**F**) Transformant producing YFP:TubB2 (strain YFP:TubB2-15-3). (**G**) Transformant producing RanGAP1:YFP (strain RanGAP1:YFP-15-1). (**H**) Transformant producing YFP:DRP1 (strain YFP:DRP1-15-8).

Supplemental Figure S3.

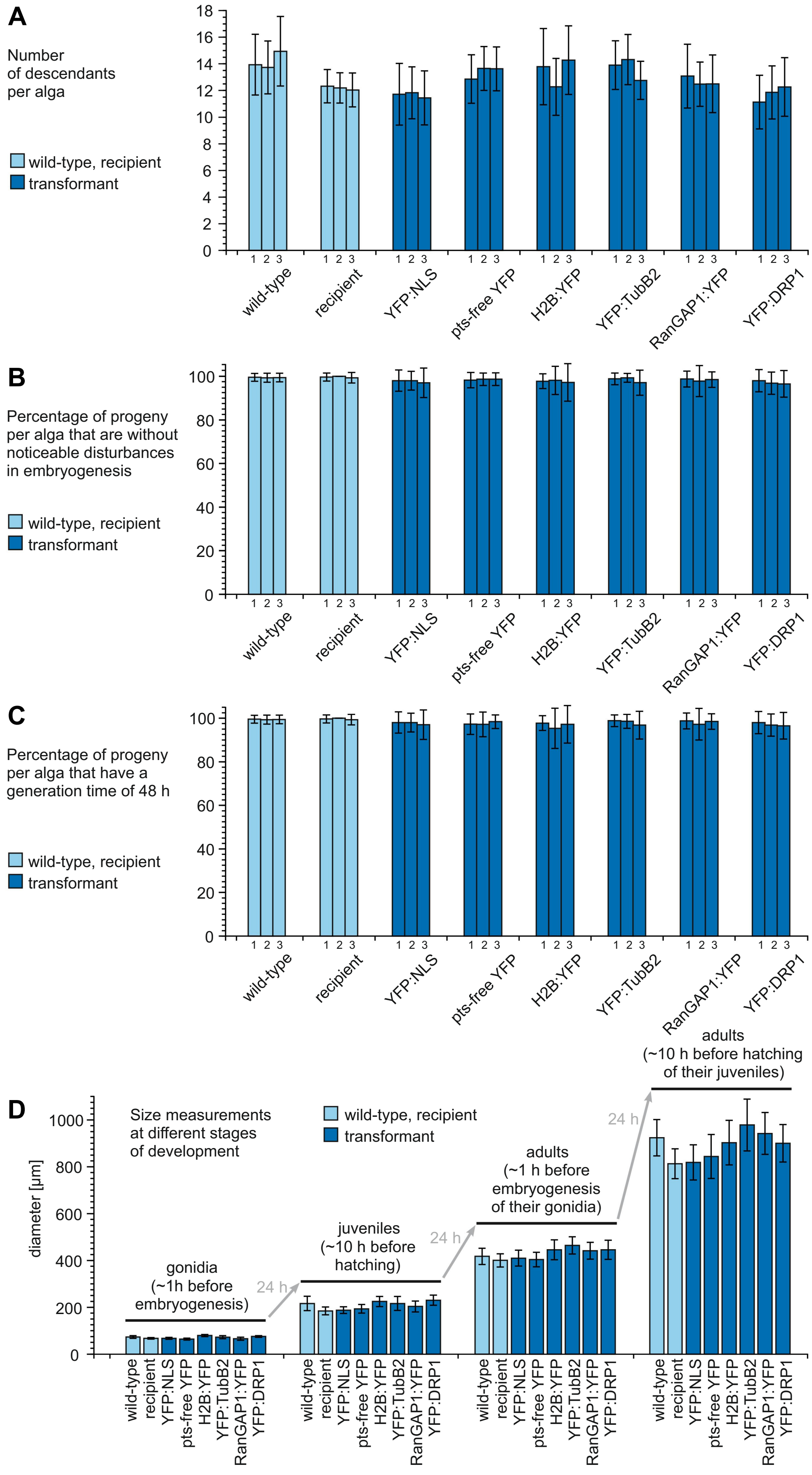

Analysis of growth behavior of wild-type, recipient and transformant *V. carteri* strains. Supports Figures 2 to 11. (**A**) Number of descendants per alga. (**B**) Percentage of progeny per alga that are without noticeable disturbances in embryogenesis. (**C**) Percentage of progeny per alga that have a generation time of 48 h. (**D**) Size measurements at different stages of development. Diameter of gonidia approximately 1 h before embryogenesis, juveniles approximately 10 h before hatching, adults approximately 1 h before embryogenesis of their gonidia and adults approximately 10 h before hatching of their juveniles. Under investigation were wild-type strain Eve10, the nitrate-reductase deficient recipient strain TNit-1013 and transformants producing YFP:NLS (strain YFP:NLS-8-1), pts-free YFP (strain pts-free-YFP-2-2), H2B:YFP (strain H2B:YFP-2-2), YFP:TubB2 (strain YFP:TubB2-15-3), RanGAP1:YFP (strain RanGAP1:YFP-15-1) and YFP:DRP1 (strain YFP:DRP1-15-8). Bars in A to C represent the means of three independent cultures per strain with an average of 27.5 +/- 3 algae. In D an average of 29 specimens were measured per parameter and strain. Error bars indicate the standard deviation.

Supplemental Figure S4.

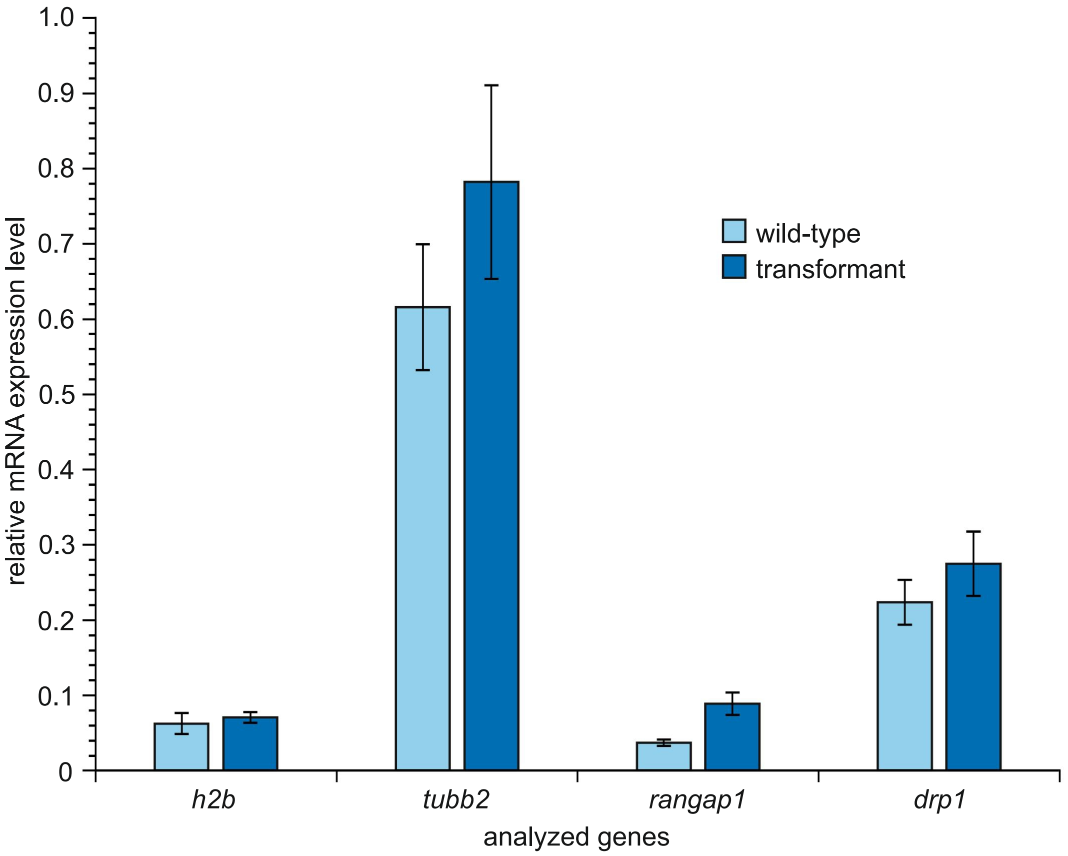

mRNA expression analysis of target genes in wild-type and transformant *V. carteri* strains. Supports Figures 5 to 11. Comparison of the mRNA expression levels of the analyzed genes in wild-type algae with those in the respective transformants. The mRNA quantification was done by RT-qPCR. The expression of the genes *h2b*, *tubb2 rangap1* and *drp1* was analyzed in wild-type algae (Eve10) to determine the natural expression level. Furthermore, *h2b* expression was analyzed in H2B:YFP transformants (strain H2B:YFP-2-2), *tubb2* expression in YFP:TubB2 transformants (strain YFP:TubB2-15-3), *rangap1* expression in RanGAP1:YFP transformants (strain RanGAP1:YFP-15-1) and *drp1* expression in YFP:DRP1 transformants (strain YFP:DRP1-15-8). In each case, the used PCR primers match both the mRNA of the naturally present gene as well as the mRNA of the gene fusions with *yfp* that were additionally integrated into the genome. The presented expression levels are means of three biological replicates (independent cultures) and normalized to the reference gene *eef1*. Error bars indicate the standard deviation.

Supplemental Figure S5.

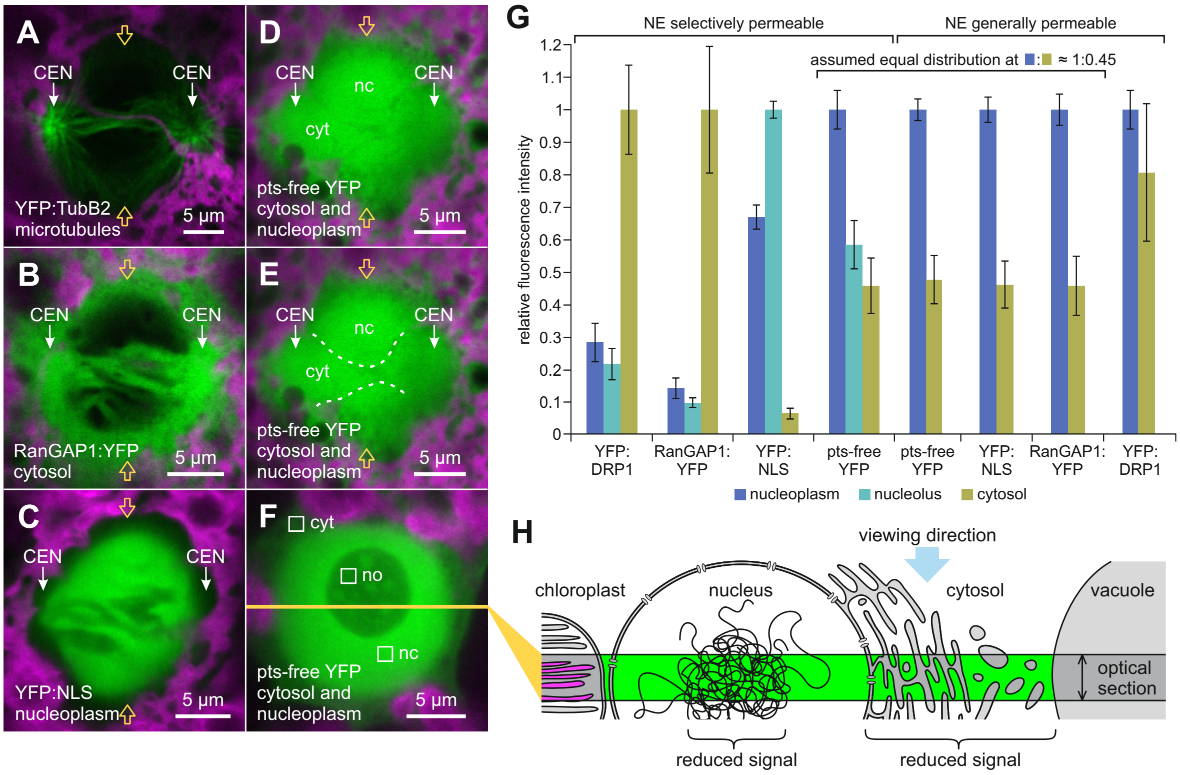

Distribution of YFP fusion proteins and pts-free YFP between cytosol and nucleoplasm. Supports Figures 2 to 11. Localization of YFP-fluorescence in transformants that produce YFP:TubB2, RanGAP1:YFP, YFP:NLS, YFP:DRP1 or pts-free YFP with a focus on the distribution of fluorescence between the cytosol and nucleoplasm considering the influences of unequally distributed cell components. (**A** to **F**) *In-vivo* CLSM imaging of YFP fluorescence (green) and chlorophyll fluorescence of chloroplasts (magenta). Top view onto gonidial nuclei during late prophase. In **A** to **E** the focal plane is at the anterior pole of the nucleus and thus contains the two separating centrosomes, which are connected by numerous microtubule strands. In **F** the focal plane goes through the center of the nucleus. Two orange arrows indicate the position of the division plane. The name and subcellular localization of the respective fluorescent protein are indicated in the lower left corner. CEN, centrosome; nc, nucleoplasm; cyt, cytosol; no, nucleolus. (**A**) Localization of YFP:TubB2 at the centrosomal asters and the antiparallel microtubules connecting centrosomes during centrosome separation. (**B**) Localization of RanGAP1:YFP in the cytosol, especially in the areas surrounding the two centrosomes and the microtubular structures between the centrosomes. (**C**) Localization of YFP:NLS in the nucleoplasm. The nuclear envelope shows negative imprints of the microtubular centrosome connection, which appear as dark areas. (**D**) Localization of pts-free YFP. Due to the impressions, the nuclear envelope appears as dark stripes in the center of the image. Cytosolic and nucleoplasmic fluorescence have about the same intensity, which is consistent with the absence of membrane-enclosed organelles in this area. (**E**) Same as in (**D**) but with dashed lines highlighting the approximate position of the negative imprint caused by the microtubular centrosome connection. (**F**)

Localization of pts-free YFP. An orange horizontal line indicates the position of the cutting line for schematic cross section in the z-direction shown in **H**. In this part of the cell, the fluorescence in the cytosol appears weaker than in the nucleoplasm. (**G**) YFP fluorescence measurements in transformants with a focus on the distribution of fluorescence between the cytosol, nucleoplasm and nucleolus before and after general permeabilization of the nuclear envelope (NE). CLSM images were analyzed during prophase (NE selectively permeable) and during prometaphase, when the nucleolus was disintegrated and the distribution of fluorescent proteins reached an equilibrium between the nucleoplasm and cytosol (NE generally permeable). For determination of mean fluorescence intensity, representative regions of interest were selected as depicted in **F**. Where appropriate, transmission-PMT images were used to determine the position of the nucleolus. Fluorescence intensity was measured in triplicates for each analyzed image and the results were normalized to the mean fluorescence intensity of the brightest compartment of each data set. The columns represent the normalized mean of three to eight different gonidia. Error bars indicate the standard deviation. (**H**) Schematic cross section at the position shown in **F** to illustrate the influences of cell components onto the measured fluorescence intensity with the example of pts-free YFP (green). Fluorescent proteins can be displaced within a given cellular area by other contents of the same area (e.g., by chromatin in the nucleolus) or excluded from an area by a barrier (e.g., the membranes of chloroplast, vacuole or endoplasmic reticulum). The resulting distribution of fluorescent proteins explains the reduced fluorescence signal in the optical section.

Supplemental Figure S6.

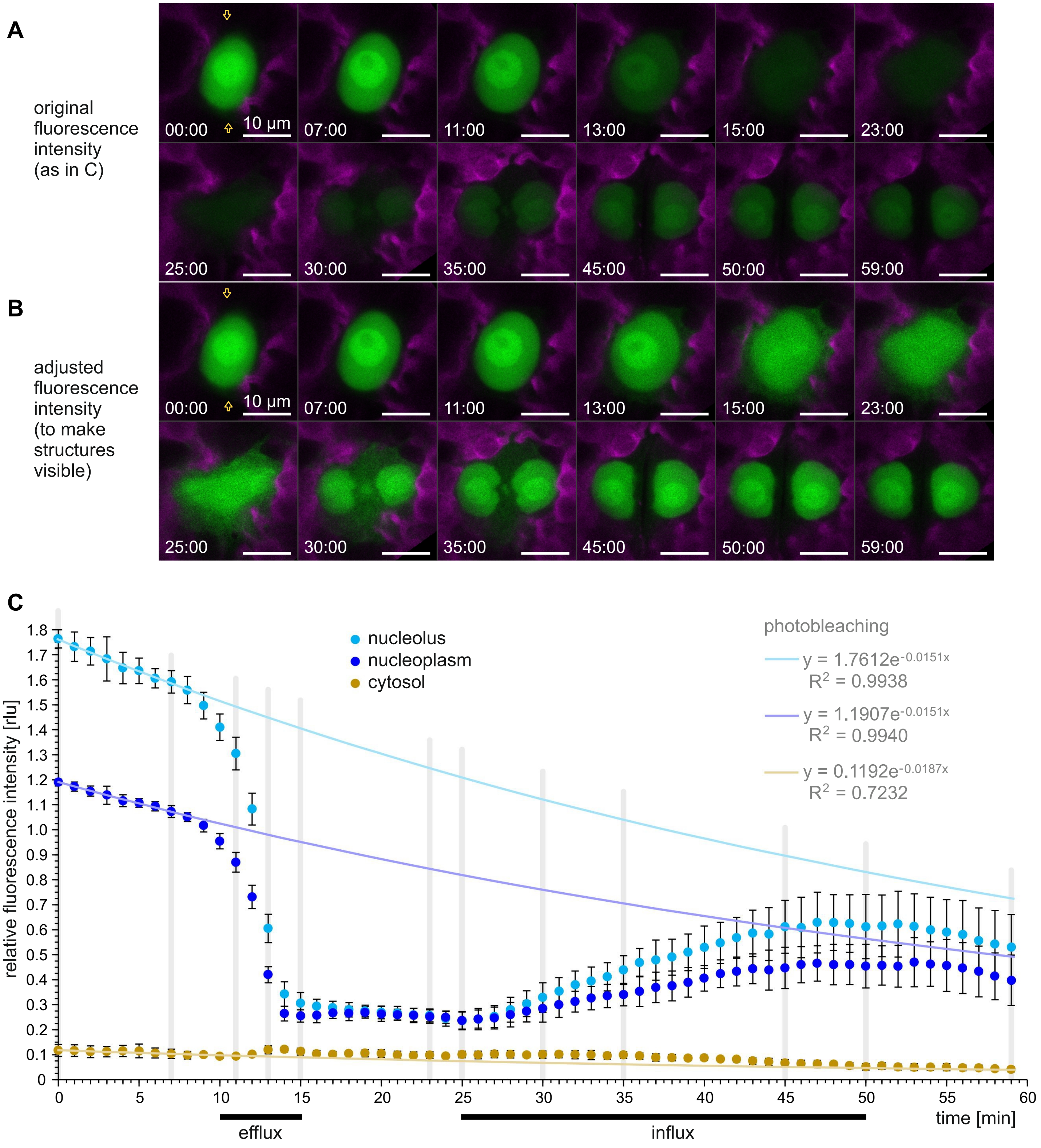

Dynamics of nuclear efflux and influx of YFP:NLS. Supports Figure 3. *In-vivo* CLSM analysis of the first embryonic cell division using *Volvox* transformants producing YFP:NLS. Time series analysis of the nuclear efflux and influx of YFP:NLS. (**A**, **B**) CLSM imaging of YFP:NLS (green). The chlorophyll fluorescence (magenta) is shown for orientation. All images are top views onto the anterior poles of gonidia. The time specifications in the lower left corners correspond to the time specifications in **C**. Two orange arrows indicate the position of the division plane. (**A**) CLSM scans with original fluorescent intensity, i.e., as shown in **C**. (**B**) Same CLSM scans as in A, but the fluorescence intensity was adjusted to make the structures visible in each image. (**C**) Time series plot showing changes in distribution of YFP:NLS fluorescence over time. ROIs were selected as shown in Figure 3A and normalization was performed as described for Figure 3. The plots represent mean values from measurements in three independent gonidia. Error bars indicate the standard deviation. The time periods for both efflux from the nucleus and influx into the new nuclei are marked. The decrease of fluorescence intensity due to photobleaching was approximated by exponential regression lines using the first nine points in time.

Supplemental Figure S7.

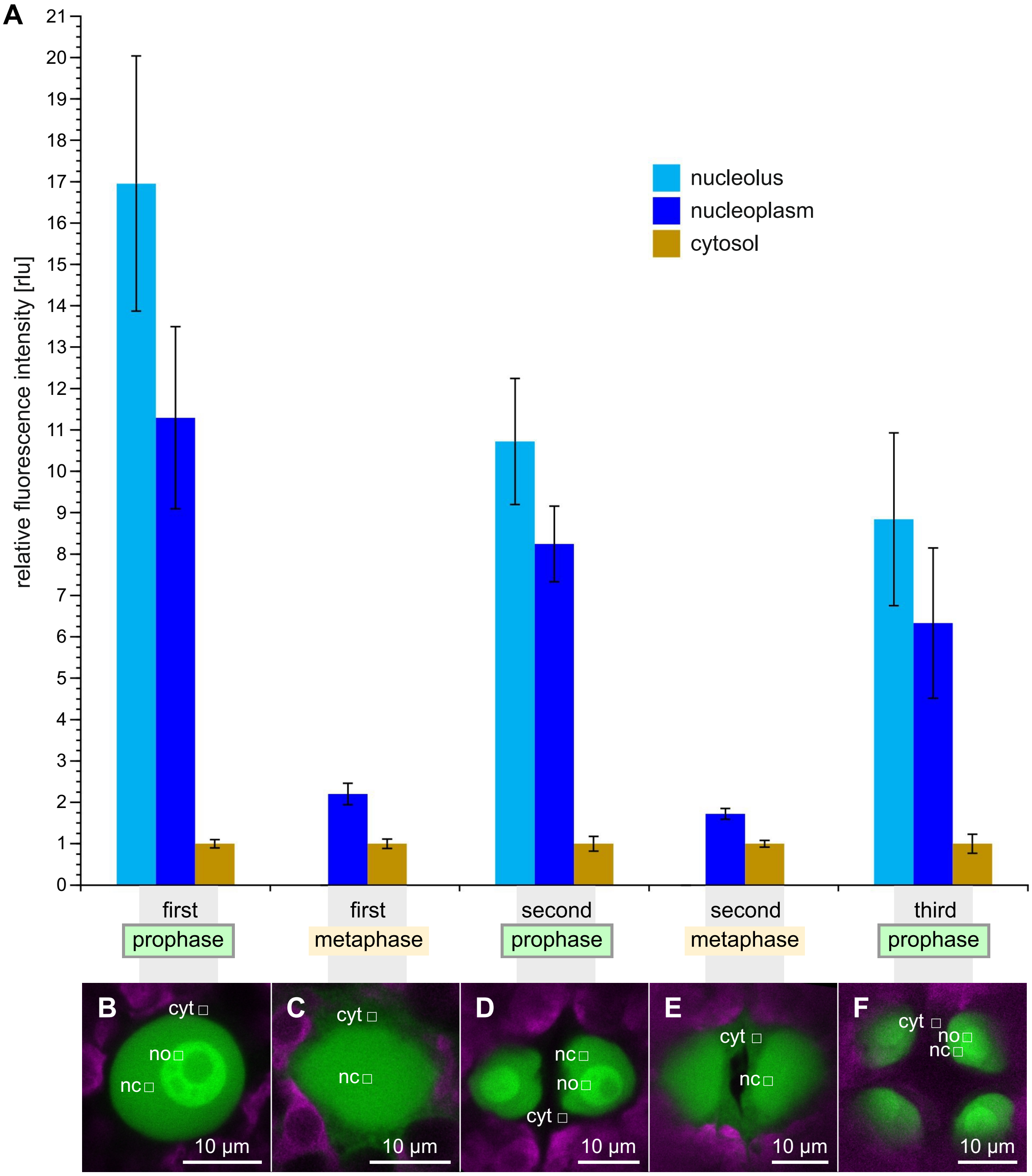

Repeated nuclear efflux and influx of YFP:NLS. Supports Figure 3. *In-vivo* CLSM analysis of the first three embryonic cell divisions using *Volvox* transformants producing YFP:NLS. (**A**) YFP fluorescence measurements showing the distribution of fluorescence between cytosol, nucleoplasm and nucleolus at prophase and metaphase of the first divisions. For determination of mean fluorescence intensity, representative ROIs of 10 x 10 pixels were selected as depicted (white squares) in **B** to **F**. Fluorescence intensity was measured in triplicates for each analyzed image and the results were

normalized to the mean fluorescence intensity of the cytosolic ROIs. The columns represent the mean of six different gonidia or embryos. Error bars indicate the standard deviation. nc, nucleoplasm; cyt, cytosol; no, nucleolus. (**B** to **F**) *In-vivo* CLSM imaging of YFP fluorescence (green) and chlorophyll fluorescence of chloroplasts (magenta). Top view onto dividing nuclei. (**B**) First prophase. (**C**) First metaphase. (**D**) Second prophase. (**E**) Second metaphase. (**F**) Third prophase.

Supplemental Figure S8.

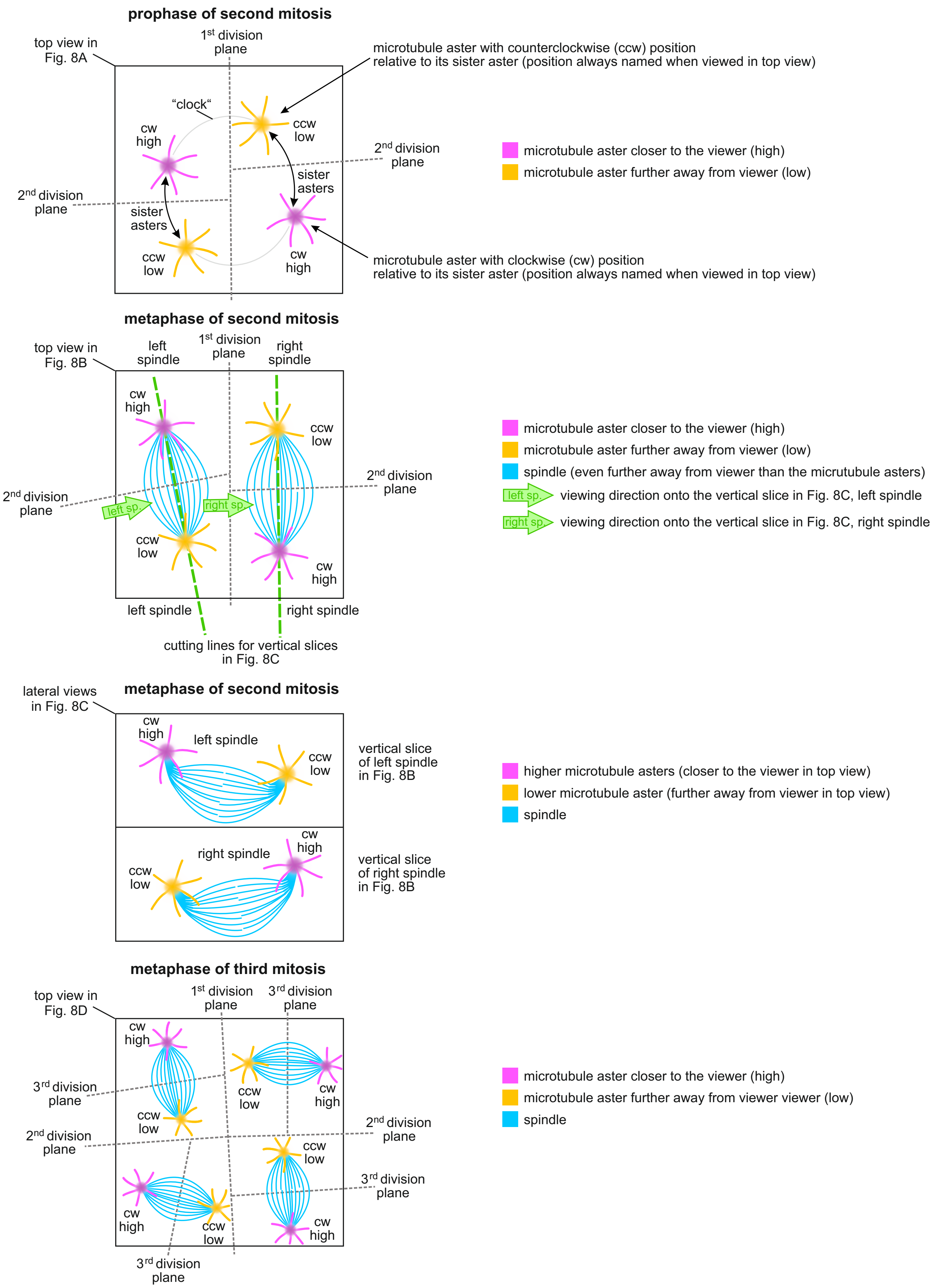

Schematic representation of microtubule asters and spindles during the second and third embryonic cell divisions. Supports Figures 8A to 8D.

Supplemental Figure S9.

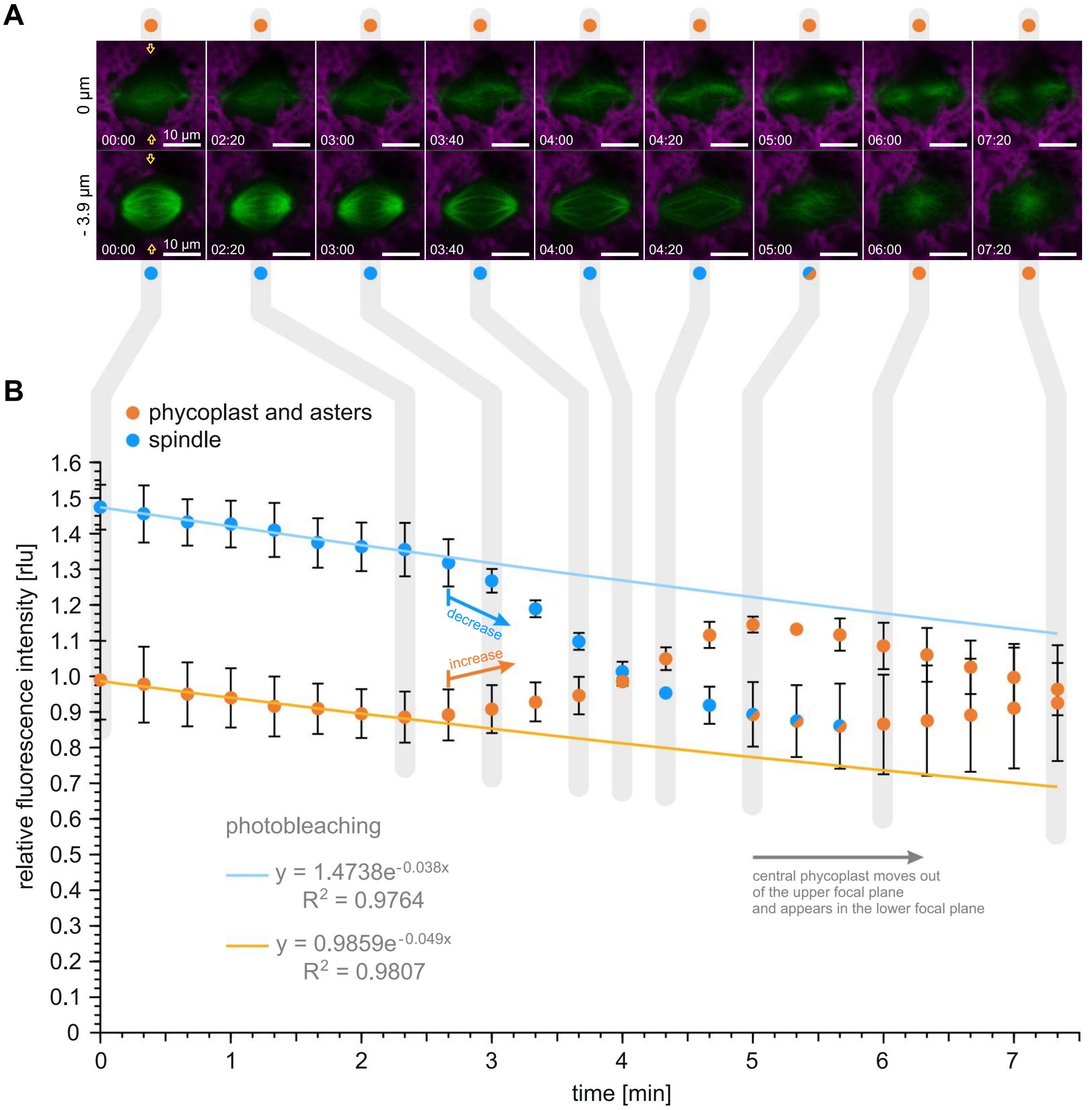

Dynamics of the microtubule cytoskeleton during spindle degradation and phycoplast construction. Supports Figures 7 to 9. Investigation of microtubular structures of dividing gonidia from metaphase to cytokinesis. Time-series analysis from two focal planes. The distance between the two focal plains is 3.9 µm. The time difference in relation to the first image is given in min:s. (**A**) *In-vivo* CLSM imaging of the first embryonic division of *Volvox* transformants producing YFP:TubB2. Top view onto a dividing gonidium showing spindle degradation and phycoplast construction. YFP:TubB2 fluorescence is displayed in green and chlorophyll fluorescence of chloroplasts is shown in magenta. Two orange arrows indicate the position of the division plane. (**B**) Time series plot showing changes in distribution of YFP:TubB2 fluorescence over time. Fluorescence intensity was measured in two focal planes: One plane contains mainly the spindle and the other plane contains mainly the phycoplast and the microtubule

asters. Mean fluorescence intensity was determined for each of the 388 x 388 pixel images. Measurements were performed in three independent cells. Time scales were aligned according to the time point at which fluorescence in the phycoplast plane exceeds fluorescence in the spindle plane (crossing point). The fluorescence intensity of the images was normalized by the mean intensity at the crossing point for each data set. The mean and standard deviation of the fluorescence intensities were then determined for each time point. Error bars indicate the standard deviation. The decrease of fluorescence intensity due to photobleaching was approximated by exponential regression lines using the first eight data points shown.

Supplemental Figure S10.

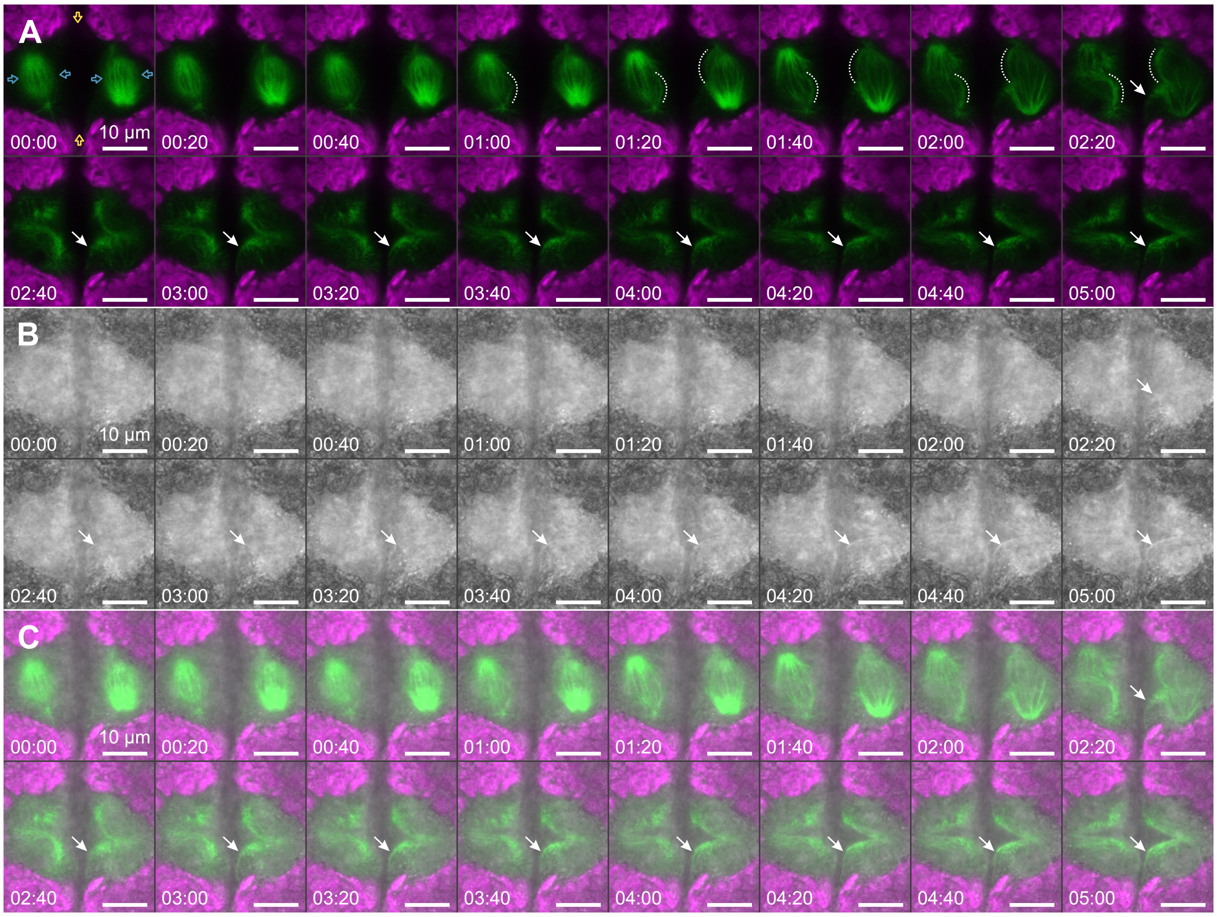

Time-series images of microtubular structures and membrane invagination of the cleavage furrow during the second cell division. Supports Figure 8E. The figure shows 16 images from the same time series of which 8 images are shown in Figure 8E. For all 16 fluorescence images, the simultaneously acquired transmission-PMT images as well as overlays are shown. (**A**) *In-vivo* CLSM imaging using *Volvox* transformants that produce YFP:TubB2. The time series starts at metaphase, ends with the initiation of the cleavage furrow during cytokinesis and shows the reorganization of spindle tubulin into microtubular structures of the phycoplast. Dotted lines mark some of the emerging hook-like structures of the phycoplast. Top view onto the microtubular structures of dividing embryos. YFP:TubB2 is shown in green and chlorophyll fluorescence of chloroplasts in magenta. Arrows indicate the approximate position of the first (orange) and second (blue) division plane. (**B**) Transmission-PMT images taken simultaneously with the time series shown in **A**. (**C**) Overlay of fluorescence images and transmission-PMT. (**A** to **C**) A white arrow serves as a reference point, thus illustrating that the formation of the phycoplast, visible by YFP:TubB2 in CLSM imaging, appears to be immediately followed by the cell membrane invagination of the cleavage furrow, which is visible in the transmission-PMT. Time is in min:s. All scale bars are 10 µm.

Supplemental Figure S11.

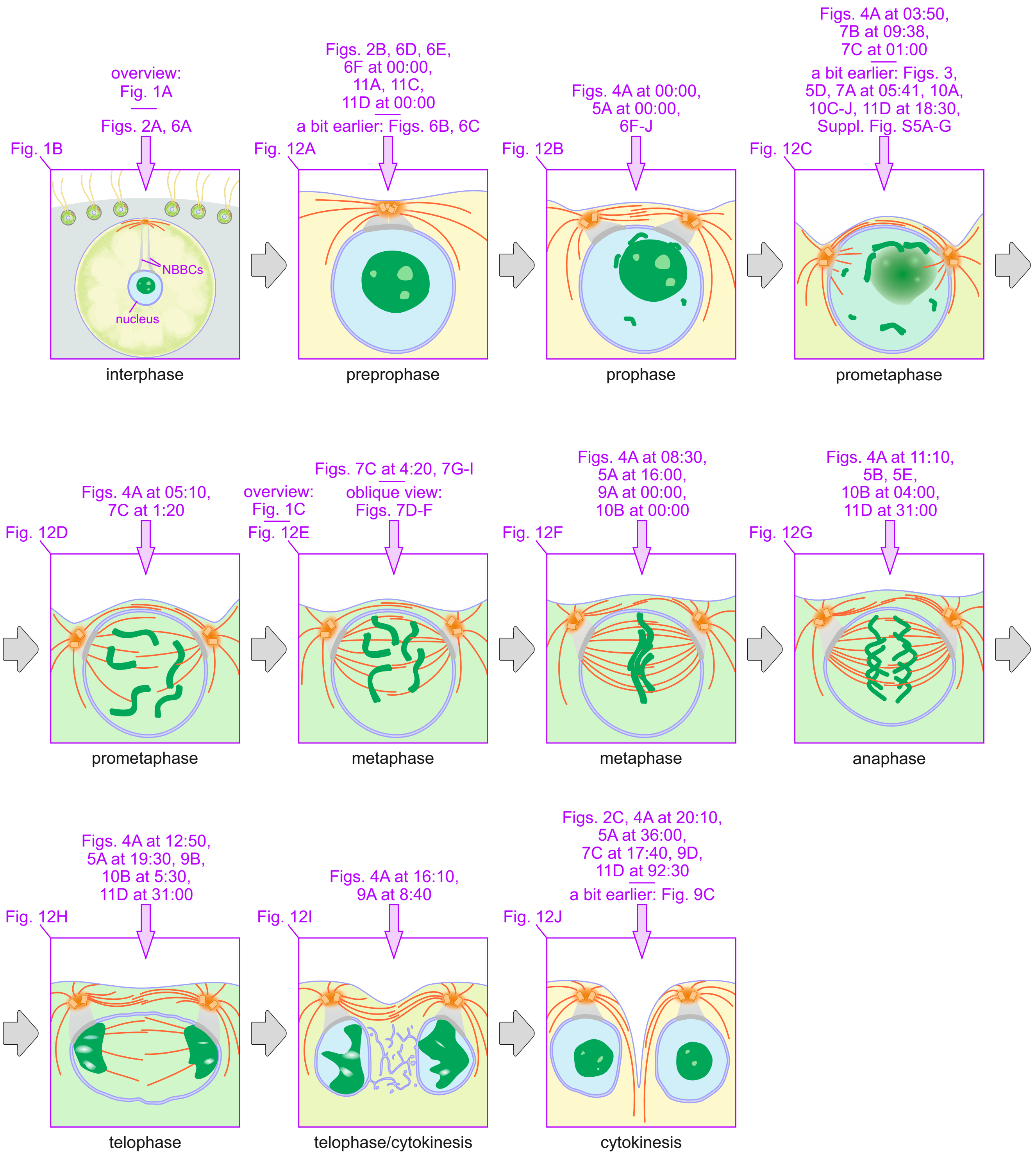

Assignment of the various figures to the individual steps of the first embryonic mitosis in *V. carteri*. Supports Figures 1 to 12. Figures indicated above the lilac vertical arrow are top views of the anterior poles of gonidia or embryos. Schematic side views are indicated by frames. The top view is the only direction with an unobstructed view of the structures shown.

Supplemental Figure S12.

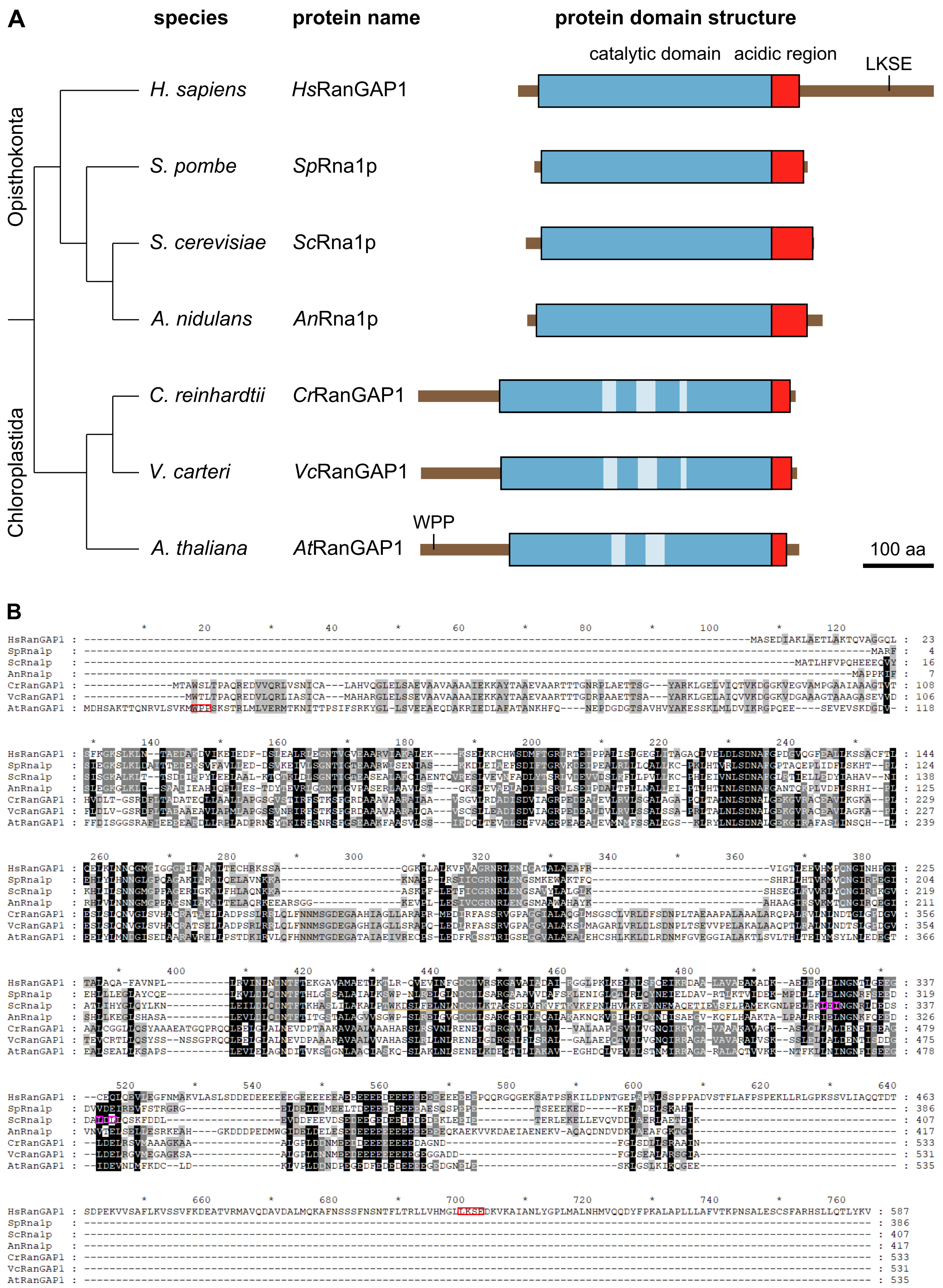

RanGAP1 proteins of several model organisms with their phylogenetic relations, structure of their RanGAP1 domains and sequence alignment of the corresponding RanGAP1 proteins. Supports Figure 10. **A** RanGAP1 proteins of several model organisms with their phylogenetic relations (left) and schematic structure of their RanGAP1 domains (right). The positions of the specific amino acid motifs WPP and LKSE are indicated. All RanGAP1 proteins contain a catalytic domain (blue) and an acidic region (red). The catalytic domains of Chloroplastida contain additional sequences (light blue), which are not present in the Opisthokonta. The RanGAP1 proteins differ particularly in their N- and C-terminal sections (brown). Chloroplastida possess an N-terminal WPP-like domain, which not always includes the sequence motif WPP. WPP-like domains are not found in Opisthokonta. Within the Opisthokonta, the metazoan RanGAP1 proteins are SUMOylated at their C-terminal sections, which requires an LKSE motif. Phylogenetic relations of the specified model organisms were taken from previous publications (Hallmann, 2011; Leonard and Richards, 2012; Kuramae et al., 2006). **B** Amino acid sequence alignment of the RanGAP1 proteins shown in **A**. Conserved amino acid residues are shaded, with similarity groups enabled. White letters on black background represent residues that are conserved in 70 to 100% of the sequences at the corresponding position, residues that are shown as white letters on dark gray background are conserved in 55 to 69% of the sequences, and residues with black letters on light gray background are conserved in 40 to 54% of the sequences. The motifs WPP and LKSE are framed in red. Residues that are potentially involved in the nuclear import or export of *Sc*Rna1p are marked in orange or magenta, respectively. The species-specific identifiers of RanGAP1 proteins are: NP_001304859 (*Homo sapiens*), NP_191872 (*Arabidopsis thaliana*), Vocar.0048s0050 (*Volvox carteri*), Cre11.g479250 (*Chlamydomonas reinhardtii*), 1K5D_L (*Schizosaccharomyces pombe*), KAF4002269 (*Saccharomyces cerevisiae*), CBF76601 (*Aspergillus nidulans*). A text file of the alignment is provided in Supplemental Data Set 5.

Supplemental Table S1. Overview of replications in terms of coverage of the mitotic phases sorted by the fluorescent protein used.

Mitotic phase	CLSM images of independent gonidia/embryos	CLSM images shown in this publication
**pts-free YFP during first embryonic division**		
prophase	3	Figure 4A at 00:00
prometaphase	3	Figure 4A at 2:10 to 5:10
metaphase	3	Figure 4A at 08:30
anaphase	3	Figure 4A at 11:10 to 12:50
telophase	3	Figure 4A at 13:10 to 20:10
cytokinesis	3	Figure 4A at 20:10 to 22:30
**H2B:YFP during first embryonic division**		
prophase	3	Figure 5A at 00:00
prometaphase	3	Figure 5A at 04:00 to 06:00
metaphase	5	Figure 5A at 10:00 to 16:00
anaphase	6	Figure 5A at 17:00 to 18:40
telophase	4	Figure 5A at 19:30 to 36:00
**YFP:TubB2 during first embryonic division**		
interphase	14	Figure 6A
preprophase	6	Figure 6B
prophase	6<br>16	Figure 6F<br>Figure 6G-J
prometaphase	19	Figure 7A-C
metaphase	21	Figure 7C at 02:40 to 07:20
anaphase	21	Figure 7C at 07:40 to 09:00
telophase	17	Figure 7C at 09:00 to 16:00
cytokinesis	24	Figure 7C at 16:00 to 20:20
**YFP:TubB2 during second embryonic division**		
prophase	6	Figure 8A
metaphase	16	Figure 8B
anaphase	19	Figure 8E at 00:00 to 01:40
telophase	19	Figure 8E at 02:00 to 02:40
cytokinesis	18	Figure 8E at 02:20 to 05:00

**RanGAP1:YFP during first embryonic division**		
prophase	13	Figure 10A at 00:00
prometaphase	13	Figure 10A at 00:15 to 01:35
metaphase	12	Figure 10B at 00:00
anaphase	11	Figure 10B at 03:30 to 05:30
telophase	9	Figure 10B at 06:00 to 12:00
cytokinesis	6	Figure 10B at 12:00 to 16:00
**YFP:DRP1 during first embryonic division**		
preprophase	8	Figure 11A and 11D at 00:00
prophase	6	Figure 11D at 17:00
prometaphase	6	Figure 11D at 19:30
metaphase	5	Figure 11D at 22:30
anaphase	5	Figure 11D at 29:00
telophase	6	Figure 11D at 30:00 to 36:30
cytokinesis	9	Figure 11D at 33:30 to 42:30

Supplemental Table S2. Primers for construction of expression vectors. Restriction enzyme sites are indicated in bold, start and stop codons are underlined. Kozak sequences, pentaglycine interpeptide bridges (Gly5) and nuclear localization signals (*NLS*) are in italics.

Amplicon name	Amplicon length	Template	Considerable sequence elements	Primer name	Primer sequence (5'→3')
lhcbm1 promoter region	1001 bp	*V. carteri* gDNA	*Xho*I*	LHCBM1_prom_for	5′-AATCCATCCCATTACTGTCAG
			*Cla*I, *Xho*I	LHCBM1_prom_rev	5′-T**ATCGAT**G**CTCGAG**CACAAGCTGGAAGTGGGAAC
lhcbm1 terminator region	309 bp	*V. carteri* gDNA	*Xba*I, Stop	LHCBM1_term_for	5′-A**TCTAGA**TAAATTCCTGGTGGAGTCATTG
			*Not*I	LHCBM1_term_rev	5′-A**GCGGCCGC**CGAGCCCGCATACATTGT
tubb2 promoter region	477 bp	*V. carteri* gDNA	*Xho*I	TubB2_prom_for	5′-A**CTCGAG**CTAGAGTTTGTAGGTAAACAGA
			*Xho*I	TubB2_prom_rev	5′-A**CTCGAG**TAAAGTCCGGAGATCACGAG
pts-free *yfp*	774 bp	plasmid pOpt_ YFPnointron_ Paro**	*Cla*I, Kozak, ATG	YFP_for	5′-T**ATCGAT***GCAACC*ATGAGCAAGGGCGAGGAGC
			*Xba*I, Stop	YFP_rev	5′-A**TCTAGA**TTACTTCTCGAACTGCGGGTG
5'-yfp	750 bp	plasmid pOpt_ YFPnointron_ Paro**	*Cla*I, Kozak, ATG	YFP_for	5′-T**ATCGAT***GCAACC*ATGAGCAAGGGCGAGGAGC
			*Cla*I, Gly5	YFP_rev_Gly5	5′-T**ATCGAT***GCCACCGCCTCCGCC*CTTGTACAGCTCGTCCATG
3'-yfp	783 bp	plasmid pOpt_ YFPnointron_ Paro**	*Xba*I, Gly5	YFP_for_Gly5	5′-A**TCTAGA***GGCGGAGGCGGTGGC*ATGAGCAAGGGCGAGGAGC
			*Xba*I, Stop	YFP_rev	5′-A**TCTAGA**TTACTTCTCGAACTGCGGGTG
yfp + *NLS*	759 bp	plasmid pOpt_ YFPnointron_ Paro**	*Cla*I, Kozak, ATG	YFP_for	5′-T**ATCGAT***GCAACC*ATGAGCAAGGGCGAGGAGC
			*Xba*I, Stop, *NLS*	YFP_rev_nls	5′-A**TCTAGA**CTA*CACCTTGCGCTTCTTCTTGGG*CTTGTACAGCTCGTCCATG
tubb2	1894 bp	*V. carteri* gDNA	*Cla*I, ATG	TubB2_cds_for	5′-T**ATCGAT**ATGCGTGAGATCGTCCACATTCAG
			*Bam*HI, Stop	TubB2_cds_rev	5′-A**GGATCC**CTAGTTCTCCTCCTCCTCG
h2b	486 bp	*V. carteri* gDNA	*Cla*I, Kozak, ATG	H2B_cds_for	5′-T**ATCGAT***TTACAA*ATGGCCCCAAAGAC
			*Spe*I	H2B_cds_rev	5′-T**ACTAGT**GGCCGACGTGAACTTGGTG
rangap1 gDNA	1115 bp	*V. carteri* gDNA	*Cla*I, Kozak, ATG	RanGAP1_gDNA_for	5′-T**ATCGAT***TCCGCG*ATGTGGACTCTTAC
			*Spe*I, *Acc*I*	RanGAP1_gDNA_rev	5′-T**ACTAGT**TGACCCTGTTGACTGAACTG
rangap1 cDNA	1373 bp	*V. carteri* cDNA	*Acc*I*	RanGAP1_cDNA_for	5′-GAGCTGGCAATCCAAGTCG
			*Spe*I	RanGAP1_cDNA_rev	5′-T**ACTAGT**TGCAATTCCAGACCGTGCC
drp1 gDNA	2065 bp	*V. carteri* gDNA	*Cla*I, ATG	DRP1_gDNA_for	5′-T**ATCGAT**ATGGAGCGTGTGATTGGACT
			*Eco*RV*	DRP1_gDNA_rev	5′-CACCCAGCCGTGCTTGAGC
drp1 cDNA	1259 bp	*V. carteri* gDNA	*Eco*RV	DRP1_cDNA_for	5′-CTGACGAAGGTG**GATATC**ATG
			*Spe*I, Stop	DRP1_cDNA_rev	5′-T**ACTAGT**CTACATGAAAGCCGCGATTTC

* Endogenous restriction site, located downstream of the respective primer and therefore not included in the primer sequence.

** Lauersen, K. J., Kruse, O. and Mussgnug, J. H. (2015) Appl. Microbiol. Biotechnol. 99, 3491-3503.
