# Supplementary material for: Molecular and cellular dynamics of early embryonic cell divisions in Volvox carteri
Source: Plant Cell. 2022 Jan 9;34(4):1326–53. doi: 10.1093/plcell/koac004 (PMC9026201; doi:10.1093/plcell/koac004)
Supplement: koac004_Supplementary_Data [file koac004_Supplementary_Data.zip › tpc.21.00708_ArticleSupplemental Movie Legends.pdf]

Supplemental Movie Legends.

Supplemental Movie S1. Efflux dynamics of nuclear YFP:NLS at prophase of the first embryonic cell division. *In-vivo* CLSM-time series for visualization of nuclear efflux dynamics showing a *Volvox* transformant that produces YFP:NLS (green). The chlorophyll fluorescence of chloroplasts (magenta) is shown for orientation. Top view onto the anterior pole of a gonidial nucleus during disintegration of the nucleolus. Individual images are shown in Figure 3.

Supplemental Movie S2. Efflux and influx dynamics of nuclear YFP:NLS during the first embryonic cell division. *In-vivo* CLSM-time series for visualization of nuclear efflux and influx dynamics showing a *Volvox* transformant that produces YFP:NLS (green). The chlorophyll fluorescence of chloroplasts (magenta) is shown for orientation. Top view onto the anterior pole of a gonidial nucleus from prophase to cytokinesis. Individual images are shown in Figure S6.

Supplemental Movie S3. Formation of the spindle apparatus and structure of the microtubule cytoskeleton during cytokinesis visualized by YFP:TubB2. *In-vivo* CLSM-time series of the microtubule cytoskeleton during the first embryonic division using *Volvox* transformants that produce YFP:TubB2 (green). The chlorophyll fluorescence of chloroplasts (magenta) is shown for orientation. Top view onto the gonidial nucleus. The series starts with the disintegration of the nucleolus during prometaphase and ends when the cleavage furrow forms in the nuclear plane in the course of cytokinesis. Individual images are shown in Figure 7C.

Supplemental Movie S4. 3D topology of microtubule asters and spindle apparatus after their detachment during metaphase. *In-vivo* CLSM imaging using *Volvox* transformants that produce YFP:TubB2 (green). The chlorophyll fluorescence of chloroplasts (magenta) is shown for orientation. 3D projection of z-stack images shown in Figure 7D-F rotating from top view to side view and back.

Supplemental Movie S5. Microtubule-based structures of the phycoplast during the first embryonic cell division. *In-vivo* CLSM imaging using *Volvox* transformants that produce YFP:TubB2. Top view onto the phycoplast. YFP:TubB2 is shown in green and chlorophyll fluorescence of chloroplasts in magenta. Time-series images showing the

concentration of microtubule bundles between the two centrosomes of the dividing gonidium building a microtubule structure that looks like two point-symmetric hooks. The series starts at the metaphase and ends with formation of the cleavage furrow during cytokinesis. Individual images are shown in Figure 9A.

Supplemental Movie S6. Topology of microtubule asters, spindles and phycoplasts during the second embryonic cell division. *In-vivo* CLSM imaging using *Volvox* transformants that produce YFP:TubB2. Top view onto the microtubular structures of dividing embryos. YFP:TubB2 is shown in green and chlorophyll fluorescence of chloroplasts in magenta. The series starts at the metaphase, ends with initiation of the cleavage furrow during cytokinesis and shows the reorganization of spindle tubulin into microtubular structures of the phycoplast. Individual images are shown in Figure 8E.

Supplemental Movie S7. 3D topology of the phycoplast during early cytokinesis of the first embryonic cell division. *In-vivo* CLSM imaging using *Volvox* transformants that produce YFP:TubB2. Rotating 3D projection of z-stack images. YFP:TubB2 is shown in green and chlorophyll fluorescence of chloroplasts in magenta. Early stage of cytokinesis showing the microtubular network of the phycoplast at the cleavage furrow.

Supplemental Movie S8. 3D topology of the phycoplast during advanced cytokinesis of the first embryonic cell division. *In-vivo* CLSM imaging using *Volvox* transformants that produce YFP:TubB2. Rotating 3D projection of z-stack images. YFP:TubB2 is shown in green and chlorophyll fluorescence of chloroplasts in magenta. Advanced stage of cytokinesis showing the microtubular network of the phycoplast at the cleavage furrow.

Supplemental Movie S9. Localization of RanGAP1:YFP during the first embryonic mitosis. *In-vivo* CLSM imaging of RanGAP1 using *Volvox* transformants that produce fluorescent RanGAP1:YFP (green). The chlorophyll fluorescence of chloroplasts (magenta) is shown for orientation. Top view onto a dividing gonidium. The series starts with the influx of RanGAP1 into the nucleus at late prophase, shortly before the disintegration of the nucleolus, and ends with the formation of the cleavage furrow during cytokinesis. Individual images are shown in Figure 10.

Supplemental Movie S10. Localization of YFP:DRP1 during the first embryonic mitosis. *In-vivo* CLSM imaging of DRP1 using *Volvox* transformants that produce

fluorescent YFP:DRP1 protein (green). The chlorophyll fluorescence of chloroplasts (magenta) is shown for orientation. Top view onto the nucleus of a dividing gonidium. The series starts at prophase and ends with the formation of the cleavage furrow in the nuclear plane during cytokinesis. Individual images are shown in Figure 11D.
